# Impacts of industrial actions, protests, strikes and lockouts by health and care workers during COVID-19 and other pandemic contexts: a systematic review

**DOI:** 10.1186/s12960-024-00923-y

**Published:** 2024-07-02

**Authors:** Isabel Craveiro, Pradeep Kumar Choudhury, Ana Paula Cavalcante de OLiveira, Alessandra Pereira, Inês Fronteira, Raphael Chança, Giorgio Cometto, Mario Roberto Dal Poz, Paulo Ferrinho

**Affiliations:** 1 Global Health and Tropical Medicine, GHTM, LA-REAL, Instituto de Higiene e Medicina Tropical, Universidade Nova de Lisboa, Rua da Junqueira, 100, 1349-008 Lisbon, Portugal; 2 Zakir Husain Centre for Educational Studies, School of Social Sciences, Jawaharlal Nehru University, Room No. 234, New Delhi, India; 3 Instituto de Medicina Social, Universidade do Estado do Rio de Janeiro, Rua São Francisco Xavier 524, 7º Andar, Blocos D E E, Maracanã, Rio de Janeiro, RJ 20550-013 Brazil; 4 National School of Public Health, Public Health Research Centre, Comprehensive Health Research Center, NOVA University of Lisbon, Avenida Padre Cruz, 1600-560 Lisbon, Portugal; 5 Instituto Nacional de Cancer, Ministério da Saúde, Rua Marquês de Pombal, 125, Centro, Rio de Janeiro, RJ 20230240 Brazil; 6 Health Workforce Department, World Health Organization, Av. Appia 20, 1202 Geneva, Switzerland

**Keywords:** COVID-19, Public health emergencies, Strikes, Health and care workforce policy

## Abstract

**Background:**

Public health emergencies of international concern (PHEICs) as the COVID-19 pandemic and others that have occurred since the early 2000s put enormous pressure on health and care systems. This is being a context for protests by health and care workers (HCWs) because of additional workload, working conditions and effects on mental and physical health. In this paper, we intended to analyze the demands of HCWs associated with industrial actions, protests, strikes and lockouts (IAPSLs) which occurred during COVID-19 pandemic and other PHEICs; to identify the impact of these grievances; and describe the relevant interventions to address these IAPSLs.

**Methods:**

We included studies published between January 2000 and March 2022 in PubMed, Embase, Scopus, BVS/LILACS, WHO’s COVID-19 Research Database, ILO, OECD, HSRM, and Google Scholar for grey literature. Eligibility criteria were HCWs as participants, IAPSLs as phenomenon of interest occurring in the context of COVID-19 and other PHEICs. GRADE CERQual was used to assess risk of bias and confidence of evidence.

**Results:**

1656 records were retrieved, and 91 were selected for full-text screening. We included 18 publications. A system-wide approach, rather than a limited approach to institutions on strike, makes it possible to understand the full impact of the strike on health and care services. PHEICs tend to aggravate already adverse working conditions of HCWs, acting as drivers for HCWs strikes, leading to staff shortages, and financial issues, both in the North and in the Global South, particularly evident in Asia and Africa. In addition, issues related to deficiencies in leadership and governance in heath sector and lack of medical products and technologies (e.g., lack of personal protective equipment) were the main drivers of strikes, each contributing 25% of the total drivers identified.

**Conclusions:**

It is necessary to focus on the preparedness of health and care systems to respond adequately to PHEICs, and this includes being prepared for HCWs’ IAPSLs, talked much in the context of COVID-19 pandemic. Evidence to assist policymakers in defining strategies to respond adequately to the health and care needs of the population during IAPSLs is crucial. The main impact of strikes is on the disruption of health care services’ provision. Gender inequality being a major issue among HCWs, a proper understanding of the full impact of the strike on health and care services will only be possible if gender lens is combined with a systemic approach, rather than gender-undifferentiated approaches limited to the institutions on strike.

**Supplementary Information:**

The online version contains supplementary material available at 10.1186/s12960-024-00923-y.

## Background

The COVID-19 pandemic caught the world unprepared to respond in a structured way, despite warnings following previous public health emergencies of international concern (PHEICs) [1] [Severe acute respiratory syndrome (SARS-CoV 2002–2004), Middle East Respiratory Syndrome (MERS-CoV 2012–2015), Zika (2015–2016), H1N1 influenza pandemic (2009), Ebola (West African outbreak 2013–2015, outbreak in Democratic Republic of Congo 2018–2020)].

The COVID-19 pandemic triggered one of the largest global health crises in more than a century [2]. Even the world's most developed economies faced the consequences of the pandemic utterly unprepared. As of 17 May 2023, around 766.4 million people were infected with this deadly virus (SARS-CoV-2) and 6.9 million deaths had been attributed to it [WHO Coronavirus (COVID-19) Dashboard, https://covid19.who.int].

Some countries managed to contain the virus, but for most, the responses to the pandemic ranged from poor to disastrous [3].

The COVID-19 pandemic represented an additional stressor to health and care systems, aggravating already fragile systems and undermining progress towards the sustainable development goals (SDG) [4]. The pandemic specifically impacted negatively on progress towards achievement of health-related SDGs [5], in particular, target 8 of SDG 3, achievement of Universal Health Coverage (UHC), including financial risk protection, access to quality essential healthcare services and access to safe, effective, quality and affordable essential medicines and vaccines for all (https://www.un.org/sustainabledevelopment/health/). This disruption to healthcare services is greatest among low-income countries [6].

Health and care workers (HCWs) are part of the first line response facing the pandemic. Nevertheless, as there are no official global figures, casualties are likely to include a considerable sum of HCWs [3]. The pandemic indisputably has affected their personal/family life, their working conditions, their physical, mental and social well-being, and they have been documented to have a higher risk of morbidity and mortality associated with SARS-CoV-2 than the general population [7]. The feminization of the workforce is a reality (globally, around two-thirds of the total HCWs are women), and the pandemic has brought women an increasing burden of care, unpaid care and increased domestic responsibilities [8], worsened in a context of wage gaps between men and women in the health sector, as well as reported violence against women as a symptom of power imbalances [9].

As a result, HCWs in several countries resorted to industrial actions, protests, strikes and lockouts (IAPSLs). When trying to address these IAPSLs, policymakers and managers faced a vacuum of knowledge as the evidence produced from other recent PHEICs has hardly been reviewed systematically. Previous efforts to synthesize evidence and provide policy guidance to inform the decision process have been carried out by the World Health Organization (WHO) to guide “human resources for health managers and policymakers at national, subnational and facility levels to design, manage and preserve the workforce necessary to manage the COVID-19 pandemic and maintain essential health services” [10]. However, as the pandemic advances and new developments are in place (i.e., mass vaccination, new strains of the virus) knowledge needs to be updated and made available.

HCWs’ IAPSLs are an ongoing phenomenon both in the Global North as well as in the Global South. The most reported in the literature are strikes, with all the ethical issues associated with measures taken by HCWs that hamper HCWs’ “duty to patients” and deprive the population of the care they need or seek [11–13].

Apparently, there was a substantial uptick in strike actions by HCWs in several countries at some point during the pandemic (e.g., Hong Kong, Zimbabwe, United States, South Korea, Kenya, Spain, Bosnia, Peru, Myanmar) [3].

The general expectation is that HCWs’ strikes would lead to a decline in care and increase in mortality. However, several studies have suggested that when doctors go on strike, mortality rates paradoxically may fall [14].

This systematic review [15] was designed and conducted to answer to the review questions in Box 1. In this paper, we report the baseline results of the systematic review.

So that the article would not lose its relevance, the authors made a non-systematized update to the search, repeating the search expression, covering the period between March 1, 2022 and January 31, 2024. Relevant articles were added to the discussion of results.

Box 1. Review questions

1. What are the implications of HCWs’ IAPSLs on service delivery, in terms of service disruptions (patients waiting lists), increased mortality and morbidity in in-patient care facilities, temporary and/or permanent drop-out of service, particularly during COVID-19 and other PHEICs?
2. What is the impact of IAPSLs on HCWs, related to the occupations, numbers involved, and days not worked, particularly during COVID-19 and other PHEICs?
3. What are the welfare grievances (e.g., fair pay, workload, working conditions, safety, security) leading to IAPSLs, particularly during COVID-19 and other PHEICs?
4. What are the interventions adopted to address the grievances and the demands of IAPSLs, particularly during COVID-19, and other PHEICs; and how the health care workers react to grievances identified and to the interventions implemented to address them?

## Methods

This systematic review followed the PICOC (Population, Intervention/phenomenon of interest, Comparison, Outcomes, Context) structure proposed by Petticrew and Roberts [16] (Additional file 1).

The protocol for this systematic review has been registered in PROSPERO (registration PROSPERO 2022 CRD42022324115 Available from: https://www.crd.york.ac.uk/prospero/display_record.php?ID=CRD42022324115.

### Eligibility criteria

The review included studies in which participants were HCWs participating in IAPSLs in the context of the ongoing COVID-19 pandemic or other five PHEICs since 2000 (SARS-CoV from 2002 to 2004, MERS-CoV from 2012 to 2015, Zika from 2015 to 2016, H1N1 influenza pandemic during 2009, West African Ebola outbreak from 2013 to 2015, and the Ebola outbreak in the Democratic Republic of Congo from 2018 to 2020), the intervention/phenomenon of interest. Whenever mentioned comparisons related to no-intervention or other interventions were considered. Outcomes considered relevant included the impact on service delivery (e.g., service disruptions, lockouts, days of work lost, numbers and type of HCW involved, lost working days), in-patient morbidity and mortality and the interventions to address HCWs’ demands.

HCWs are defined as all health-related occupations in the health and social sectors of employment, and various settings of employment, including health facilities offering all level of care, institutional and residential care homes, amongst others [17, 18].

IAPSLs are defined as any collective withdrawal of services or work stoppage, temporary show of dissatisfaction by employees to protest against bad working conditions or low pay and to increase bargaining power with the employer and intended to force the employer to improve them by reducing productivity in a workplace, by a group of individuals that work in health facilities offering all level of care (including those involved in patient care as physicians, nurses, midwives, and pharmacists), institutional and residential care homes, amongst others [19].

A PHEIC is defined in the International Health Regulations (2005) as “an extraordinary event which is determined to constitute a public health risk to other States through the international spread of disease and to potentially requires a coordinated international response” [20].

The types of publications elected were observational (i.e., cohort and cross-sectional), experimental, quasi-experimental, reviews, mixed methods and qualitative studies published between 1st of January 2000 and 1st of March 2022 in English, French, Hindi, Portuguese, Italian or Spanish.

### Information sources and search strategy

For this review, the following scientific databases were used: PubMed, Embase, Scopus, LILACS via BVS, WHO’s COVID-19 Research Database, International Labour Organization (ILO) [21], Economic Co-operation and Development (OECD) [22] and The Health System Response Monitor (HSRM) [23] and Google Scholar.

To identify the search terms, the controlled vocabularies of the health area DeCs (Descriptors in Health Sciences), MeSH (Medical Subject Headings) and Emtree (Embase Subject Headings) were consulted. The search strategy is detailed in additional file 2.

### Selection of studies

We used Endnote [24] to collect, organize and manage references retrieved from the searches of the different database. Once this phase was completed, we uploaded the references file to Rayyan [25].

The selection using the eligibility criteria was made by two researchers independently. Differences were discussed with a third researcher until a consensus was reached.

Eligibility and exclusion criteria were first applied to titles and abstracts of the documents uploaded in Rayyan, independently by two reviewers. In case no abstract was available, the criteria were applied either to the executive summary or to the first five pages of the introduction of the document.

**Fig. 1:**
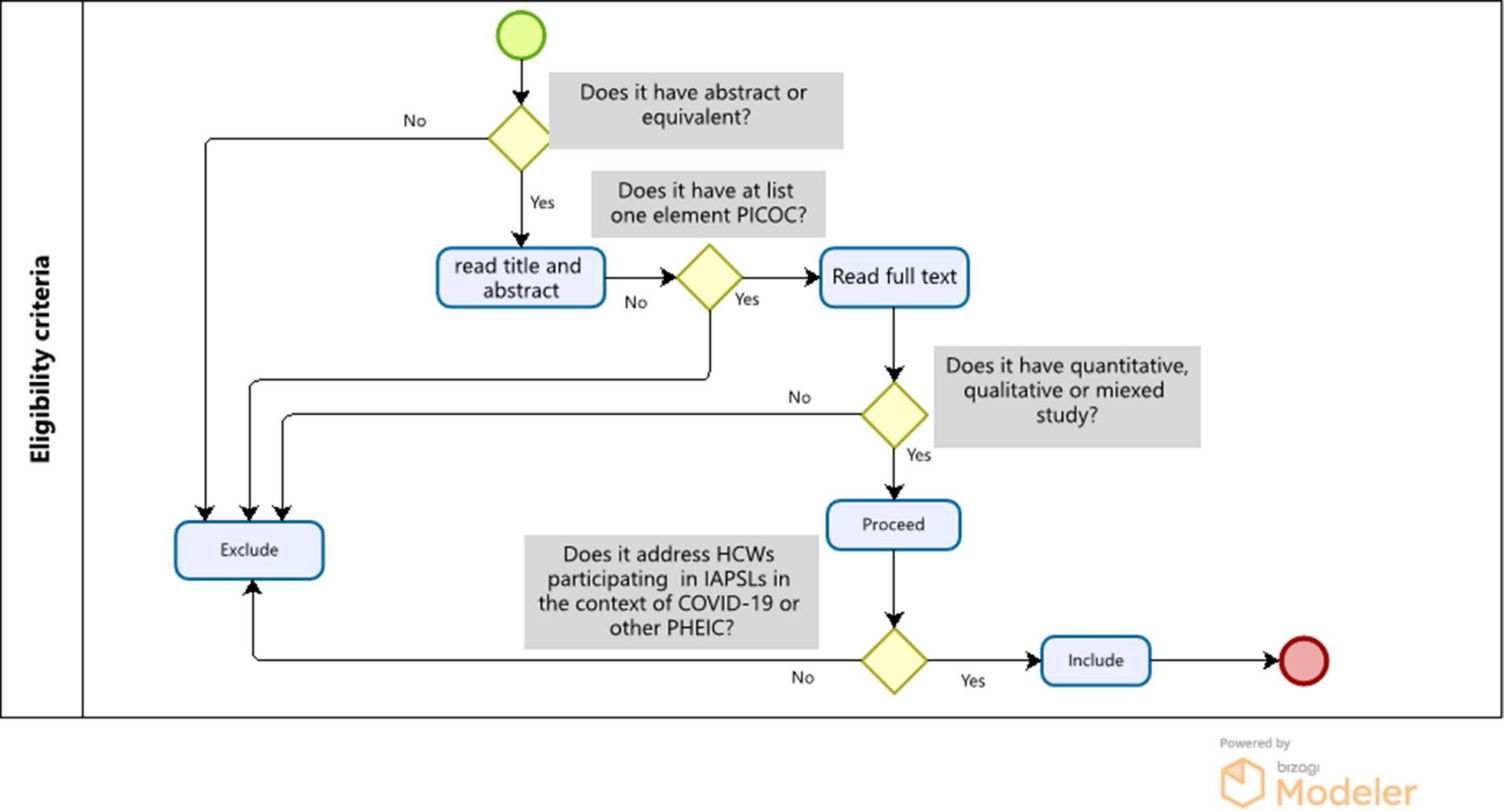
Eligibility criteria flow diagram

If eligibility criteria were met, documents were selected for full-text analysis, applying again the eligibility criteria (Fig. 1). If again fulfilled, relevant data were then extracted from the document. In case of disagreement a discussion between the two reviewers was done and a third referee reviewer was consulted. Inter-reviewer agreement was measured by estimating Kappa statistics and computing sensibility and sensitivity was computed to assess and the quality of this process [26].

**Fig. 2:**
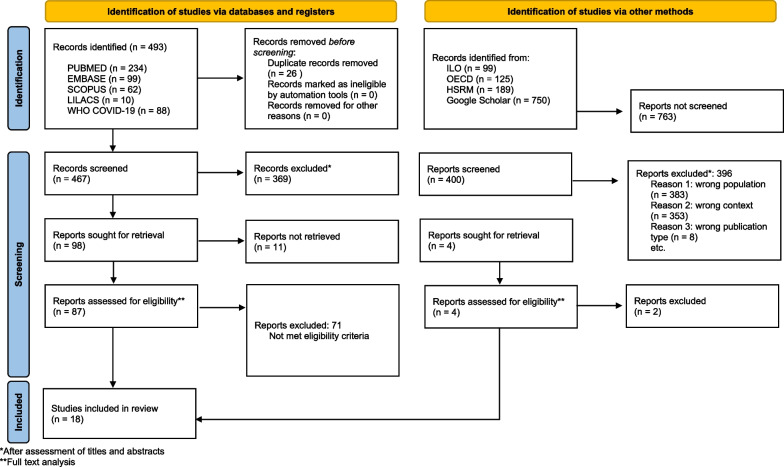
PRISMA flow chart of studies selection phase

Inclusion of publications is described as recommended by PRISMA [27, 28] (Fig. 2).

### Data extraction

Data extraction from included studies was randomly distributed between the two reviewers and was performed using an electronic form in REDCap [29]. Information extracted from selected articles included author, year, country, context, title, language, study design, and sample (Table 3—Data extraction).

### Assessment of the risk of bias of the studies

To assess the certainty of the evidence of included studies, we used an electronic form of the GRADE CERQual—Confidence in Evidence from Reviews of Qualitative research) for quantitative and qualitative studies [30] developed in REDCap and specific for the study design (Additional file 3).

The GRADE CERQual includes four components to assess the degree of confidence in the results of reviews (also called qualitative evidence synthesis): methodological limitations, coherence, data adequacy and relevance. This tool should not be seen as a mechanistic approach as confidence assessment is a subjective process [30].

### Data synthesis

Narrative synthesis was used to review and synthesize the extracted data from the systematic review. We undertook a qualitative content analysis, organizing the dimensions interconnected with the research questions, as follows: the nature and intensity of the IAPSLs (related with review question 2); the main reasons leading to HCWs’ IAPSLs (related review question 3); the results of these IAPSLs—outputs, outcomes, and impacts (related review questions 1, 2 and 4). In the analysis, there was a concern to verify the gender issues addressed in the articles.

**Table 1:** Number of publications from the first phase of the systematic review

Total	867
Included	102
Excluded	765
Reasons to exclude	
Inadequate outcome	116
Inadequate population	634
Inadequate context	681
Inadequate study design	56
Inadequate intervention	143

Many studies have more than 1 reason

**Table 2:** Inter-reviewer agreement between the two researchers

	Kappa	Standard error	C.I 95%	Agreement
K _IC/PC_	0.855	0.143	(0.574, 1.136)	Almost perfect

The kappa value was calculated for measuring agreement between two reviewers (IC and PC) for phase 1. The Kappa result should be interpreted as follows: values ≤ 0 as indicating no agreement and 0.01–0.20 as none to slight, 0.21–0.40 as fair, 0.41– 0.60 as moderate, 0.61–0.80 as substantial, and 0.81–1.00 as almost perfect agreement

**Table 3:** Data extraction about included publications

Authors/year of publication	Context (PHEIC)	Country	Title	Language	Journal	Study design	Sample (population or studies)
Erceg M, Kujundzić-Tiljak M, Babić-Erceg A, Coric T, Lang S. 2007 [31]	SARS threat*	Croatia	Physicians' strike and general mortality: Croatia's experience of 2003	English	Coll Antropol	Population study of mortality	Country census data
Wichterich C. 2021 [32]	COVID-19	India	Protection and Protest by ‘Voluntary’ Community Health Workers: COVID-19 Authoritarianism in India	English	Historical Social Research/Historische Sozialforschung	Literature and reports review	Sample not available
Adam MB, Muma S, Modi JA, Steere M, Cook N, Ellis W, Chen CT, Shirk A, Muma Nyagetuba JK, Hansen EN. 2018 [33]	Ebola threat*	Kenya	Paediatric and obstetric outcomes at a faith-based hospital during the 100-day public sector physician strike in Kenya	English	BMJ Glob Health	Cross-sectional	18 months of hospital data available
Kaguthi GK, Nduba V, Adam MB (2020) [34]	Ebola threat*	Kenya	The impact of the nurses', doctors' and clinical officer strikes on mortality in four health facilities in Kenya	English	BMC Health Services Research	Ecological survey	Data from three public and one private faith led facility
Ong'ayo G, Ooko M, Wang'ondu R, Bottomley C, Nyaguara A, Tsofa BK, Williams TN, Bejon P, Scott JAG, Etyang AO. 2019 [35]	Ebola threat*	Kenya	Effect of strikes by health workers on mortality between 2010 and 2016 in Kilifi, Kenya: a population-based cohort analysis	English	Lancet Glob Health	Population-based cohort analysis	7 year demographic surveillance data (regional)
Scanlon ML, Maldonado LY, Ikemeri JE, Jumah A, Anusu G, Bone JN, Chelagat S, Keter JC, Ruhl L, Songok J, Christoffersen-Deb A. 2021 [36]	Ebola threat*	Kenya	A retrospective study of the impact of health worker strikes on maternal and child health care utilization in western Kenya	English	BMC Health Serv Res	Cross-sectional	1571
Scanlon ML, Maldonado LY, Ikemeri JE, Jumah A, Anusu G, Chelagat S, Keter JC, Songok J, Ruhl LJ, Christoffersen-Deb A. 2021(b) [37]	Ebola threat*	Kenya	'It was hell in the community': a qualitative study of maternal and child health care during health care worker strikes in Kenya	English	Int J Equity Health	In-depth interviews and focus group discussions	73
Suiyanka L, Alegana VA, Snow RW. 2021 [38]	COVID-19	Kenya	Insecticide-treated net distribution in Western Kenya: impacts related to COVID-19 and health worker strikes	English	Int Health	Cross-sectional	25 months of health information data from 8 counties
Waithaka D, Kagwanja N, Nzinga J, Tsofa B, Leli H, Mataza C, Nyaguara A, Bejon P, Gilson L, Barasa E, Molyneux S. 2020 [39]	Ebola threat*	Kenya	Prolonged health worker strikes in Kenya-perspectives and experiences of frontline health managers and local communities in Kilifi County	English	Int J Equity Health	Interviews, focus groups Document review	34 5 studies
Aborisade R.A. & Gbahabo D.D. 2021 [40]	COVID-19	Nigeria	Policing the lockdown: accounts of police officers´ aggression and extortion of frontline health workers in Nigeria	English	Policing and Society	Interviews	62
Mayaki S, Stewart M. Teamwork, 2020 [41]	Ebola	Nigeria	Professional Identities, Conflict, and Industrial Action in Nigerian Healthcare	English	J Multidiscip Healthc	Focus groups and interviews	41
Oleribe OO, Ezieme IP, Oladipo O, Akinola EP, Udofia D, Taylor-Robinson SD. 2016 [42]	Ebola	Nigeria	Industrial action by healthcare workers in Nigeria in 2013–2015: an inquiry into causes, consequences and control—a cross-sectional descriptive study	English	Hum Resour Health	Cross-sectional	150
Oleribe OO, Udofia D, Oladipo O, Ishola TA, Taylor-Robinson SD. 2018 [43]	Ebola threat*	Nigeria	Healthcare workers' industrial action in Nigeria: a cross-sectional survey of Nigerian physicians	English	Hum Resour Health	Cross-sectional	58
Mavis Mulaudzi F, Mulaudzi M, Anokwuru RA, Davhana-Maselesele M. 2021 [44]	COVID-19	South Africa	Between a rock and a hard place: Ethics, nurses' safety, and the right to protest during the COVID-19 pandemic	English	Int Nurs Rev	Exploratory Literature review	Sample not available
Cho YH, Cho JW, Ryoo HW, Moon S, Kim JH, Lee SH, Jang TC, Lee DE. 2022 [45]	COVID-19	South Korea	Impact of an emergency department resident strike during the coronavirus disease 2019 (COVID-19) pandemic in Daegu, South Korea: a retrospective cross-sectional study	English	J Yeungnam Med Sci	Cross-sectional	31,357
Sim Jeongyong, Choi, Yuri and Jeong Jinwoo, 2021 [46]	COVID-19	South Korea	Changes in Emergency Department Performance during Strike of Junior Physicians in Korea	English	Emergency Medicine International	Cross-sectional	2,385
Murphy (2022) [47]	COVID-19	USA	Exploring the Ethics of a Nurses' Strike During a Pandemic	English	Am J Nurs	Review	Not available
Russo G, Xu L, McIsaac M, Matsika-Claquin MD, Dhillon I, McPake B, et al. 2019 [48]	Studies included cover PHEICs that occurred between 2009 and 2018	23 low-income countries	Health workers’ strikes in low-income countries: the available evidence	English	Bull World Health Organ	Systematic review	116 records

*Although the country has no reported cases, the strike took place in the context of a threat from a PHEIC

**Table 4:** GRADE CERQual assessment

Author/year	Findings	Evidence	Summary assessment
Aborisade et al. (2021)	This study was conducted to elicit accounts of health workers, sequel to the indefinite sit-at-home strike embarked upon by the Nigeria Medical Association in the middle of the COVID-19 outbreak, to protest alleged harassment and extortion of its members during the lockdown by police officers. Occasionally, these aggressive and extortive behaviours were specifically targeted at health workers based on officers’ impressions and prior unpleasant experiences with health institution and its personnel. These findings have important policy and practical implications, if the physical health and emotional wellbeing of frontline health workers are to be recognised and met, in supporting them in their role of combating COVID-19	Moderate confidence	Interviews were performed using snowball sampling. The analysis is explained but not deeply discussed. The researchers don´t present any reflexion on the study, namely about methodological limitations The data addresses the phenomenon of interest (strikes by HCWs), is coherent because the results relate to the phenomenon of strikes by health professionals in the context of COVID-19, and relevant as the results contribute to understanding the strikes by health professionals in an area usually not covered (the intersections between policing and public health in PHEIC context)
Adam et al. (2018)	The study analyzes patient admissions and deaths at the AIC-Kijabe Hospital, a not-for profit, faith-based Kenyan hospital, before, during and after the 100-day physician strike The volume of patients increased and exceeded the hospital’s ability to respond to needs. There were increases in deaths during the strike period across the paediatric medical, newborn, paediatric surgical and obstetric units with an OR (95% CI) of death of 3.9 (95% CI 2.3–6.4), 4.1 (95% CI 2.4–7.1), 7.9 (95% CI 3.2–20) and 3.2 (95% CI 0.39–27), respectively. Increased mortality across paediatric and obstetrical services at AIC-Kijabe Hospital correlated with the crippling of healthcare delivery in the public sector during the national physicians’ strike in Kenya	Moderate confidence	The period of analysis is justified to consider the analysis of data before, during and after the physician strike and therefore understand the impact of the phenomenon in the outcome (mortality and patient admissions). The finding reflects the data, and the analysis is appropriated. But the article has a limited analysis to a single hospital. The authors acknowledge that the increase in strike-related mortality may reflect a referral bias Although the country has no reported Ebola cases, the strike took place in the context of a threat from a PHEIC
Cho et al. (2022)	This article investigates the impact of a 19-day resident strike during the COVID-19 pandemic on the mortality and ED LOS of patients who visited the EDs of six teaching hospitals in Daegu, South Korea. Doctors do not strike frequently because of ethical concerns During the study period, 31,357 patients visited the ED, of which 7749 belonged to the experimental group. Control periods 1 and 2 included 13,100 and 10,243 patients, respectively. No significant in-hospital mortality differences were found between strike periods, however, the results showed statistically significant differences in the length of ED stay. Both the length of ED stays and the number of patients visiting the ED decreased	High confidence	The method is clearly explained, and the researchers used appropriate statistical analysis. There is a noticeable coherence between phenomenon of interest, data and findings. The authors of the study acknowledge that examining the impact of a strike on patients only based on mortality is a limited approach, and additional studies are needed to develop more accurate measurement approaches. The findings are relevant to understand the phenomenon of interest
Erceg et al. (2007)	This study analysed mortality data from the National Bureau of Statistics relating to the strike period (15 January–14 February 2003) were selected and compared with the previous and subsequent periods of the same duration in 2001, 2002 and 2004. Of the 52,575 deaths in 2003, Croatia recorded 4,682 (8.9%, 95% Confidence interval 8.4–9.4) in the strike period from the 15th of January to the 14th of February 2003 or 1.1 deaths per 1000. No deviations of the 15th of January to the 14th of February period’s share of the death total in relation to other observation periods were noted. It is impossible to associate the strike based on the figures shown in this paper with either an increase or decrease in population mortality	High confidence	Method is clearly explained, analysis details are provided. There is no limitation in the methodology, except for the absence of researchers’ reflexivity The data are clear and coherent, addressing the phenomenon of interest (impact of strikes by HCWs on mortality), although the country has no reported Sars cases, the strike took place in the context of a threat from a PHEIC, so the findings might be considered relevant to better understand the overall phenomenon of interest
Kaguthi et al. (2020)	The study assessed the mortality impact of a 100-day physician strike which was followed by 151-day nurses’ strike and 20-day clinical officer strike in Kenya. There was a significant decline in the numbers of patients seen, comparing the non-strike and strike periods; beta (ß) coefficient − 649 (95% CI − 950, − 347) p < 0.0001. The physicians’ strike saw a significant decline in mortality (ß) coefficient − 19.0 (95% CI − 29.2, − 8.87) p < 0.0001. Nurses and Clinical Officer strikes did not significantly impact mortality. There was no mortality increase in the post-strike period beta (ß) coefficient 7.42 (95% CI − 16.7, 1.85) p = 0.12	Moderate confidence	The method is clearly explained, the rational for the purposeful selection of four health facilities was justified, and the researchers used appropriate statistical analysis. Researchers recognize the limitations of using an ecological study, namely because it is not possible to assess whether the patients who used the health facilities during the strike were different in any way from the non-strike period. Although the country has no reported Ebola cases, the strikes took place in the context of a threat from a PHEIC, and the findings contribute to better understand the phenomenon under research in the review
Mavis et al. (2021)	This paper critically discusses the ethics of nurses’ choice to strike during the COVID-19 pandemic, considering legal and ethical arguments, overlaying the Ubuntu philosophy, an African ethic. Nurses thus may be justified in striking to protect their safety. State healthcare entities are obliged to ensure safety and protect the health of professionals during the pandemic. According to their Code of Practice and Pledge of Service, they are ethically obliged to put patients first, and as a result, they are legally barred from engaging in strike action	Moderate confidence	The study presents methodological limitations, lacking detail on data collection and analysis, but the researchers included a reflexion on methodological limitations and their impact on review findings as well as justification of analysis. The study is coherent regarding phenomenon of interest but is not possible to assess the ambiguity. The study was developed during COVID-19 pandemic. The ethical discussion is very pertinent to understand the phenomenon of interest justifying the relevance of the study
Mayaki et al. (2020)	This study discusses the complexities of factors perceived to cause workplace conflicts, including the extent to which these are thought to link to industrial action Reasons for incessant industrial actions: poor leadership; remuneration Barriers to teamwork include professional hierarchy, role ambiguity, and poor communication	High confidence	The study is methodologically correct, including a section on researcher reflectivity and the topic is very relevant. It is well designed, the selection of participants is clear, the collection and analysis of data is adequate The study is coherent as the findings include HCWs industrial actions that occurred during Ebola, findings are clear. The findings reflect the perspectives of HCWs on the causes of industrial actions (lacking the information of their participation in the industrial actions) but reflect the motivations of trade unions leaders to embark on industrial actions. The theme is relevant and well-developed
Murphy (2022)	This study discusses the ethical dimension of nurses strikes during a pandemic such as COVID-19, considering that the ethical calculus (that is, finding the right balance between beneficence and harm before deciding on a course of action) must take account of a confluence of factors, including the risks to present patients, future patients, and health care workers; the severity and duration of the risks; and the availability of ameliorative or protective steps that reduce risk and harm. The principle of beneficence to both future patients and health care workers may be thwarted if the risk analysis is confined only to short-term concerns (that is, to concerns occurring within a narrow temporal window). If a significantly elevated risk has been demonstrated to affect nurses and other health care workers of colour disproportionately, racial justice must also be considered. The purpose of this article is to assess the moral framework of a work stoppage by nurses during a pandemic	Low confidence	Details on methodology are absent, and the researcher don´t present any reflexion on the study, namely about methodological limitations The coherence is established as the study was developed during COVID-19 pandemic contextualising the phenomenon of interest and analysing the ethical dimensions of nurses´ strikes in general but exploring the specificities of the USA. The ethical discussion is very important to understand the phenomenon of interest justifying the relevance of the study
Oleribe et al. (2016)	The purpose of this study was to identify the root cause(s) of strikes by healthcare workers, their effects on the health system and possible solutions to prevent, or at least reduce, industrial action. Less than half of the participants (43.6%) supported industrial actions. Poor healthcare leadership and management were cited as the most common (92%), as well as the most important (43.3%), cause of healthcare worker strikes in Nigeria. Other causes cited were a demand for higher salaries and wages (82%), infrastructural issues (63.3%) and inter-personal issues (61.3%). Only 2.0% rated current healthcare management as excellent, while 24.0% rated it as very good. Several strategies were cited towards improving healthcare management	Moderate confidence	The method was a cross-sectional descriptive study using a self-administered pre-tested structured questionnaire with open-ended questions, and the convenience sampling strategy was not justified. The sample population for the study was limited to one state of the country, and the sample size of 150 health workers may not have been adequate to power the study to allow for generalization of the findings to a national level. The researchers present a reflexion on the study, namely on methodological limitations. The analysis is appropriated There is coherence between findings, data, and phenomenon of interest. Although data are adequate, the sample size should have been increased The data addresses the phenomenon of interest, so the findings are relevant to better understand the HCWs motivations to adhere to and/or promote industrial action during PHEICs
Oleribe et al. (2018)	The study documents physicians’ views on healthcare worker-initiated strike action in Nigeria. Poor staff welfare was cited by 16.7% as the commonest cause of strikes in the healthcare system. Other causes cited were salary issues (13.9%), leadership and management (13.9%), poor hospital infrastructure (11.1%), poor guidelines and services (11.1% each) and inter-professional disputes (5.6%). The negative consequences of strikes, the groups who benefit from them and solutions to the strikes were enumerated, including training physicians in leadership skills by 98.2% of respondents	Moderate confidence	The method was a cross-sectional descriptive study using a self-administered pre-tested structured questionnaire with open-ended questions, and the convenience sampling strategy used justified to ensure inclusion of participants from all the geopolitical regions of the country. The sample is small as well as the response rate (44.3%). The analysis is appropriated. The researchers present a reflexion on the study, namely on methodological limitations The data addresses the phenomenon of interest, and the strike took place in the context of a threat from Ebola, so the findings might be considered relevant to better understand the overall phenomenon of interest
Ong'ayo et al. (2019)	The study assessed the effect on mortality of six strikes by health workers that occurred from 2010 to 2016 in Kilifi, Kenya Between Jan 1, 2010, and Nov 30, 2016, 1,829,929 person-years of observation, 6396 deaths, and 128 strike days (median duration of strikes, 18.5 days [range 9–42]) were recorded In the primary analysis, no change in all-cause mortality was noted during strike periods (adjusted rate ratio [RR] 0.93, 95% CI 0.81–1.08; p = 0.34) Weak evidence was recorded of variation in mortality rates by age group, with an apparent decrease among infants aged 1–11 months (adjusted RR 0.58, 95% CI 0.33–1.03; p = 0.064) and an increase among children aged 12–59 months (1.75, 1.11–2.76; p = 0.016). No change was noted in mortality rates in post-strike periods and for any category of cause of death	Moderate confidence	The method is clearly explained, and the researchers used appropriate statistical analysis. An added value of the study is the use of demographic surveillance data obtained over 7 years, although the analysis is not nationwide. The findings reflect the impact of WCWs strikes that took place in the context of a threat from a PHEIC. Although the country has no reported Ebola cases, these findings are useful to understand the overall phenomenon, so the findings might be considered relevant. The delimitation of the period of analysis and the data gathering approaches were adequately explained
Russo et al. (2019)	Of a total of initial 676 records, 109 met the inclusion criteria after elimination of duplicates based on customized Google searches. In total, 116 reports covering 70 unique strike episodes in low-income countries met our inclusion criteria. Of the reports identified, most (103) were online media reports, five human resources for health reports from ReliefWeb and The World Bank databases and two academic publications The identified records report health workers’ strikes in 23 low-income countries during the period, accounting for 875 days of strike. Year 2018 had the highest number of events (17), corresponding to 170 workdays lost. Strikes involving more than one professional category was the frequent strike modality (32 events), followed by strikes by physicians only (22 events). The most reported cause was complaints about remuneration (63 events), followed by protest against the sector’s governance or policies (25 events) and safety of working conditions (10 events). Positive resolution was achieved more often when collective bargaining institutions and higher levels of government were involved in the negotiations	High confidence	The systematic review presents a clear methodology only lacking researchers’ reflexivity. It covers the phenomenon of interest (strikes of HCWs). Although the findings don´t address directly any PHEIC, the studies included in the study cover a period (2009–2018) and territory (low- and middle-income countries) where public health emergencies of international interest occurred. It is clear, rich in details, and presents a framework useful for future studies on the subject
Scanlon et al. (2021)	This study assessed the impact of nationwide strikes by health workers in 2017 on utilization of maternal and child health services in western Kenya. Women in the strike group were 17% less likely to attend at least four ANC visits during their pregnancy (ARR 0.83, 95% CI 0.74, 0.94) and 16% less likely to deliver in a health facility (ARR 0.84, 95% CI 0.76, 0.92) compared to women in the control group. Whether a child received their first oral polio vaccine did not differ significantly between groups, but children of women in the strike group received their vaccine significantly longer after birth (13 days versus 7 days, p = 0.002)	Moderate confidence	The study recruited a cohort of pregnant women in 2017 and a different cohort of pregnant women in 2018 when there were no major strikes by health workers. The method was clear and explained in detail, the researchers present some level of reflexivity. The data have coherence, and the findings reflect the phenomenon of interest. Although the country has no reported Ebola cases, the strike took place in the context of a threat from a PHEIC The study is limited in terms of geographic coverage as presents data from only one County in Kenya
Scanlon et al. (2021)(b)	Participants reported that strikes by health care workers significantly impacted the availability and quality of maternal and child health services in 2017 and had indirect economic effects due to households paying for services in the private sector. Participants felt it was the poor, particularly poor women, who were most affected since they were more likely to rely on public services, while community health volunteers highlighted their own poor working conditions in response to strikes by physicians and nurses. Strikes strained relationships and trust between communities and the health system that were identified as essential to maternal and child health care	Low confidence	Methodological detail is presented but the selection criteria were not mentioned. The authors present a reflexion on the findings. There are limitations in method replication for achieving a strong impact. The study is limited in terms of geographic coverage because it includes participants from only one county in Kenya Although the study addresses the phenomenon of interest (strikes by HCWs), it addresses its impact indirectly from the perspective of users, managers and community heath volunteers. The country has no reported Ebola cases, although the strike took place in the context of a threat from a PHEIC, so the findings might be considered relevant to better understand the overall phenomenon of interest
Sim, Choi and Jeong (2021)	A total of 1121 and 1496 patients visited the ED during the strike and control periods (both 17 days), respectively. The care usually provided by four or six physicians, including one specialist, was replaced with that by one or two specialists at any one time. During the trainee doctors’ strike, EM specialists managed patients with fewer consultations. However, the proportion of patients who underwent laboratory and radiologic tests did not change significantly[i.e., median ED length of stay significantly decreased from 359 min (interquartile range, IQR: 147–391) in the control period to 326 min (IQR: 123–318) during the strike period (P < 0.001)]. The doctors’ strike was not found to have a significant effect on mortality after adjustments with other variables Fewer acute patients diverted to the appropriate level of care Although the physician workforce was reduced and the acuity of the patients increased, crude mortality did not increase during the strike The EDLOS in the study period was significantly decreased, and the difference in median EDLOS was 33 min. Similarly, the median time to disposition orders decreased by 30 min	High confidence	The study is methodologically consistent, with a solid approach and the topic is very relevant. The study has no methodological limitations. It is well designed, the selection of participants is clear, the collection and analysis of data is in accordance with best practices. Statistical analysis is adequate. The method directly impacts the findings The study is coherent as the results relate to the phenomenon of HCWs strikes in the context of COVID-19, the results are clear and there is no ambiguity in the data The study is detailed, but not nationwide, restricted to a single hospital
Suiyanka et al. (2021)	The median monthly distribution of LLINs declined during COVID mitigation strategies (March–July 2020) and during the health worker strikes (December 2020–February 2021). Recovery periods followed initial declines, indicative of a ‘catching up’ on missed routine distribution. Mass community campaigns were delayed by 10 months. These observations offer encouragement for routine net distribution resilience, but complete interruptions of planned mass distributions require alternate strategies during pandemics	Low confidence	The analysis presented in the study is not nationwide (information data from only 8 counties), there are lack of detail on statistical analysis performed, and no reflectivity of researchers is presented. Tools were adequate for this kind of research. The study is coherent because the results relate to the phenomenon of strikes by health professionals in the context of COVID-19, and relevant as the results contribute to understanding the impacts of strikes by health professionals in an area usually not covered (e.g., reduction in the distribution of mosquito nets in a territory with a high prevalence of malaria and therefore with important needs for activities to control the vector)
Waithaka et al. (2020)	The study explores the perceptions and experiences of frontline health managers and community members of the 2017 prolonged health workers’ strikes In the face of major health facility and service closures and disruptions, frontline health managers enacted a range of strategies to keep key services open, but many strategies were piecemeal, inconsistent and difficult to sustain. Interviewees reported huge negative health and financial strike impacts on local communities, and especially the poor. There is limited evidence of improved health system preparedness to cope with any future strikes	Low confidence	Interviews and focus group discussions were performed but detail on inclusion criteria and selection of participants are lacking. Documents were also selected and reviewed. The analysis is not clearly explained neither deeply discussed. The researchers present a reflection on the study, namely about methodological limitations The data addresses the phenomenon of interest (strikes by HCWs), it addresses its impact indirectly from the perspective of PHC workforce health managers and community members, although the country has no reported Ebola cases, the strike took place in the context of a threat from a PHEIC, so the findings might be considered relevant to better understand the overall phenomenon of interest
Wichterich (2021)	Having gained confidence in earlier struggles, ASHAs complained about extreme exhaustion, increased vulnerability, and the depletion of caring capacities. Their efforts reflect a feminisation of labour struggles that focuses on care work with an emphasis on both the care-recognition-gap and the care-pay-gap. Transferring responsibility, risks, and workloads to ASHAs during the COVID-19 pandemic reflected political practices of doing vulnerability at the intersection of gender and caste Although ASHAs appeared to have gained bargaining power from state and community dependence on their services, they were unable to change the structures of care extractivism or the risk externalisation outside the formal public health sector. This situation mirrors a new type of labour struggle and organising namely the feminisation of protests—an emphasis on care work, and the implied care-recognition gap and care-pay gap	Moderate confidence	In this review of printed reports, newspapers, and journals, we consider that there are moderate concerns regarding methodological limitations because methodology was poorly described, no details on data collection and analysis methods were provided, and the researcher presents some level of reflexivity The study present findings about a category of HCWs poorly addressed in the literature and add a different perspective on the phenomenon of interest. Nonetheless, the verification of potential interpretations bias was not possible The report presents a detailed discussion that consider a national perspective allowing a better understanding of the phenomenon of interest, therefore we have moderate concerns on data adequacy. No or very minor concerns about relevance because the report refers to the current topic of COVID-19, covering both setting and population relevant to the review

**Table 5:** Geographical dimension and duration of the strikes

Country	Duration	Geographical extension	HCWs involved	Associated PHEIC*	Reference
Croatia	30 days	Nationwide	Physicians	SARS threat**	Erceg et al. [31]
India	2 days	Nationwide	Community health workers	COVID-19	Wichterich [32]
Kenya	3–7 days	Different locations	Several categories of HCWs	COVID-19	Suiyanka, Alegana, and Snow [38]
	20 days	Nationwide	Public sector middle-level HCWs, mid-level physician–assistant clinicians	Ebola threat**	Scanlon et al. [36] Scanlon, Maldonado, Ikemeri et al. [37] Adam Muma, Modi et al. [33] Waithaka, Kagwanja, JNzinga et al. [39] Kaguthi, Nduba, Adam [34]
	100 days	Nationwide	Public sector physicians		
	150 days	Nationwide	Public sector nurses		
	128 strike days - Six strikes between 2010 and 2016	Different locations	Physicians and nurses		Ong’ayo, Ooko, Wang’ondu et al. [35]
Nigeria	6 strike actions between April 2016 and April 2017	Local, state and regional; mostly in southern regions	Physicians	Ebola threat***	Oleribe et al. [43] Mayaki and Stewart [41] Oleribe et al. [42]
	8 general strikes lasting a total of 36 months (2012–2015)	All government tertiary hospitals nationwide + Local strikes at individual institutions	Public HCWs		
	8 general strikes lasting a total of 36 months (2012–2015)	national, regional and state-based strikes	Physicians, nurses and allied healthcare workers		
	Indefinite sit-at-home strike from 20 May 2020	National	Physicians	COVID-19	Aborisade and Gbahabo [40]
South Africa	General ethical discussion	NA	Nurses	COVID-19	Mulaudzi, et al. [44]
South Korea	17 days	Nationwide	Interns and residents	COVID-19	Sim Jeongyong, Yuri Choi, Jinwoo Jeong [46]
	19 days	Nationwide	Residents and specialist physicians	COVID-19	Cho, Y. H. et al. [45]
United States of America	“limited duration”	Several states	Nurses	COVID-19	Murphy [47]

**Although the country has no reported cases, the strike took place in the context of a threat from a PHEIC

***The country reported Ebola, the article analyzes the phenomenon of interest in the context of a threat from Ebola

**Table 6:** Drivers of HCWs strikes during public health emergencies of international concern (PHEICs)

Country	Nature of the IAPSLs	HCWs involved	Years	Associated PHEIC	Building block related to the demands driving the IAPSLs					Reference
					Leadership/governance	Financing	Workforce	Medical products and technologies	Service delivery	
Croatia	Strike	Physicians	2003	SARS threat**						Erceg M. et al. [31]
India	Strike	Community Health and care workers	2020	COVID-19	Sense of being abandoned by the state (left alone and unprotected in the community work during lockdown)	Better payment The COVID-19 allowance (€0.37 per day) was delayed	Professional recognition; regularisation of their labour	Lack of PPE; were asked to pull their saris over their noses and mouths, or to buy their own hand sanitisers and masks	Unsafe practice conditions led to the death of thousands during first wave depletion of caring capacities	Wichterich [32]
Kenya	Strike	Public sector physicians and nurses	2010–2016	Ebola threat**		Delays in salaries, remittance of statutory deductions, and promotions have been workers’ unions demanding		Overuse of public dispensaries, health centers and all maternal health care due to the abolition of user fees in 2013, including deliveries in all hospitals Without the equivalent increase in facilities and personnel Resulted in stockouts and equipment breakdowns	Issues related to poor quality of care and ill-equipped providers and facilities increased investment in the public health sector	Ong’ayoet al [35] Scanlon et al. [36] Scanlon et al. [37] Adam et al. [33] Kaguthi et al. [34]
		Physicians	2016							
		Public sector physicians, nurses and clinical officers	2016–2017		Breaking of collective bargaining agreements by the government	Low pay	Staff shortages in public facilities			
		Health workers (different cadres)	2020–2021	COVID-19		Better payment and contracts		Safety concerns, including availability of PPE		Suiyanka et al. [38]
		Nurses, physicians	2017	Ebola threat**	Discontent with the 2013 devolution of healthcare services -devolution process was rushed resulting in challenges with human resource management functions broader political context & activities took precedence over negotiation with nurses as politicians and government focused on national and county elections	Government’s failure to honour agreements with unions simmering discontent with human resource processes differences and unfairnesses across cadres of health workers		Poor working conditions		Waithaka et al. [39]
Nigeria	Industrial action	All categories	2012–2015	Ebola	Leadership of health sector	Remuneration issues	Inter-professional disputes			Mayaki and Stewart [41]
		Physicians and nurses						Infra-structural issues		Oleribe et al. [42]
	Strike	Physicians	April 2016 and April 2017	Ebola threat***	Lack of government leadership and management capacity to implement policies	Remuneration issues (Poor workers’ compensation; Inconsistent salaries; Delayed salary payment; Paycheck is delayed and very small to meet up with present day reality; Remuneration skipping/relativity)	Poor staff welfare (Delayed promotion; Failure of the management to sponsor residents’ exams and updates; Denial of basic entitlement such as salary and training sponsorship)	Infra-structural issues (Poor hospital utility facilities e.g., no water in wards, poor/no physicians’ call room; Poor working environment Security of staff)		Oleribe et al. [43]
	Strike	Physicians		COVID-19	Divergent directives being given to police officers in enforcing the lockdown (conducive to violence against HCWs)				Harassment (police aggression and extortion) during the lockdown,	Aborisade and Gbahabo [40]
South Africa	Took State to court	Nurses’ union	2020	COVID-19	Demand ability to participate in policy making			Lack of PPE	Unsafe practice conditions	Roelf & Winning, 2020 cit in Mavis Mulaudzi et al. [44]
	Strike									
South Korea	Strike	Residents and interns	2020	COVID-19	Protest over the plan regarding a medical school for public health					Sim Jeongyong, Yuri Choi, Jinwoo Jeong [46]
	Strike	Residents and specialist physicians	2020	COVID-19	Opposition to the government’s healthcare policies					Cho et al. [45]
United States of America	Strike	Nurses	2020–2021	COVID-19			Inadequate staffing of many hospitals and health care facilities Lack of training in infection control practices (particularly in nursing homes)	Lack of PPE Infrastructures inadequate architectural design (especially regarding airflow and isolation capacity)		Murphy [47]

** Although the country has no reported cases, the strike took place in the context of a threat from a PHEIC

*** The country reported Ebola, the article analyzes the phenomenon of interest in the context of a threat from Ebola

In total, 493 studies from databases were identified. After the exclusion of duplicates (*n* = 26), 467 references remained, and 1163 publications were identified from grey literature but only the first 100 publications from each information source were screened (*n* = 400). First, after reading the title and abstract, 102 studies were select for retrieval (98 from databases and 4 from grey literature). Then, 91 studies were selected for full text reading (87 from databases and 4 from grey literature). We identified 18 publications, all in English, which met the inclusion and quality criteria for data extraction (14 studies and 4 other publications). The entire selection process is documented in Fig. 2.

The summary of the total number of articles, conflicts between the 2 researchers for sensibility test, reasons for exclusions and included articles is presented in Table 1.

The inter-reviewer agreement, according to Kappa standard, was calculated and is shown in Table 2.

## Results

1656 records were retrieved, duplicates were checked, and the screening inclusion criteria were successively applied (in the different phases as shown in the flowchart), until the final phase in which we were left with 91 documents that were selected for full text screening (87 from the databases data and 4 studies identified via other methods). We included 18 publications after assessment for eligibility, ranging from 2007 to 2022. Ten were cross sectional studies, 4 were qualitative studies and 4 were reviews.

Fourteen country-specific publications met the inclusion and quality criteria for data: 11 were from Africa (7 from Kenya, and 4 from Nigeria); 2 from Asia (South Korea), 1 from Europe (Croatia). Four review articles were also included: 1 focused on several low-income countries, 1 from South Africa, 1 from India, and one with a focus on the United States of America (USA). Most of the publications referred to IAPSLs by physicians and/or nurses (*n* = 12).

Details on data extracted from the publications included in the review are presented in Table 3.

The findings from the records, outcomes and details of GRADE’s assessment are shown in Table 4. The evidence in the studies ranged from low to high confidence. The majority (14/18) of publications were rated high and/or moderate, based on details provided in the summary assessment, the findings should be with confidence.

### Nature and intensity of the industrial actions, protests, strikes and lockouts

The majority of IAPSLs addressed in the literature reviewed referred to HCW strikes. Only in two studies, the HCW protests are entitled industrial actions. Given the nature of the IAPSLs addressed in the literature review from now on we will refer to strikes rather than IAPSLs when describing our findings.

Strikes occurred in two contexts: countries where there is an entrenched environment of IAPSLs by HCWs, which has been aggravated by COVID-19 and other PHEICs; and countries where HCWs’ strikes are unusual.

Countries with an entrenched environment of strikes by HCWs, aggravated by COVID-19 and other PHEICs, include India [32] and South Korea [45, 46] in Asia and Kenya [33–39], Nigeria [40–43] and South Africa [44] in sub-Saharan Africa. In Nigeria, in the last decade, no sector has been more affected by strikes than the health sector and HCW industrial action is described as “commonplace throughout history” [42]. In South Africa, HCWs’ strikes has been particularly associated to strong nurses' activism [44].

There is a growing labour unrest also in countries not widely perceived to experience this problem [49, 50].

The most common strike modality was the one engaging more than one type of HCWs (9/18 publications) [34–39, 41, 42, 48], followed by strikes by physicians (6/18) [31, 33, 40, 43, 45, 46] by nurses in South Africa and in the USA (2/18) [44, 47] and by community health workers in India (1/18) [32] (Table 5).

Both the duration of the strikes and the geographical dimension are not homogeneous. Strikes’ duration ranged from 2 days to 36 months. In some countries, such as Nigeria and Kenya, they tend to have a recurrent nature. They could be restricted to one institution, one locality, or extend to several locations, state-wide, to several states or even countrywide (Table 5).

The literature refers mainly to Asian and African, and it is striking the absence of quality literature on the issue from other parts of the World.

### Main reasons leading to HCWs’ industrial action, protests, strikes and lockouts

In this systematic review, we intended to describe the main grievances of HCWs related to one or more of WHO’s health system building blocks [51]. Their grievances are mostly concerned with five building blocks: leadership and governance, financing, HCWs, medical products and technologies and service provision.

**Fig. 3:**
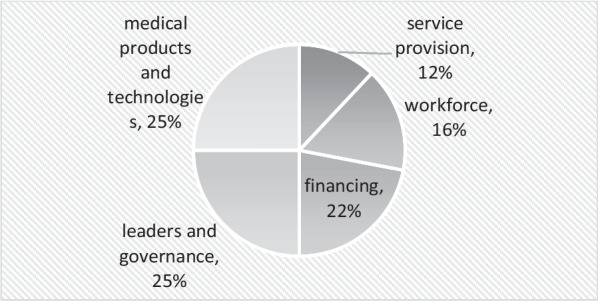
Leading causes of strikes

Many HCWs work under health and care systems with significant leadership and governance fragilities, inadequate payment systems, under-resourced with health personnel, with inadequate access to medical products and technologies (ventilators, oxygen, and personal protection equipment (PPE), providing unsafe care in sub-optimal conditions. These conditions are further aggravated during PHEICs, unprecedented by all standards but completely predictable [1]. These can be considered the main drivers of HCWs’ strikes during PHEICs (Table 6). As we can see in Fig. 3, issues related to leadership and governance and medical products and technologies were the ones that came up most frequently in the articles analyzed.

When we compare the pre-COVID-19 strikes to those that occurred during the pandemic, the protests have in common demands for better wages and payments as well as complaints about leadership decisions and contestation over healthcare policy options. The reference to the lack of PPE and harassment by security forces due to the lockdown is only mentioned in strikes during COVID-19 and strikes in this pandemic period had a greater focus on unsafe working conditions. Despite the specificities of demands of HCWs during PHEIC the common thread in all protests is related to the eventual failure of governments to maintain an operational health system and to provide HCWs with adequate means to carry out their work [52].

During the COVID-19 pandemic, the extraordinary challenges governments and health and care systems faced in creating safe environments for HCWs has also led to a spate of protests from nursing organizations throughout the world. Mavis Mulaudzi et al. [41] mentioned that the American Nurses Association, the Royal College of Nursing, and the International Council of Nurses have all registered their dissatisfaction with the limited supply of PPE and the subsequent risk this poses to nurses as health workers involved in clinical care delivery. They also refer that African nurse unions united in collective protests against inadequate working conditions, defending that African HCWs must raise their voices regarding their needs, including the provision of PPE, to inform policies during the COVID-19 pandemic [41]. “An independent analysis has identified industrial dispute and strike action in 84 countries since February 2020; of which 38% and 29% of strikes are due to poor working conditions and lack of PPE, respectively” [53].

PHEICs created a ground propitious to HCWs express their demands and manifest their protest over long-standing fragilities in several of the building blocks of the health and care systems. One example is Nigeria where one of the causes of the strikes during Ebola related to leadership and management: perceived deficiencies in the health system may have been exacerbated by the PHEIC. This is also the case for Kenya and South Korea, where the reasons for some of the IAPSLs do not seem directly linked to the concurrent PHEIC that may have served simply as a backdrop to continue protests related to long-standing grievances [39, 46].

In Kenya, there was also a different outcome for physicians’ and nurses’ demands following the 2013 strikes, which led to subsequent disputes related to promises made to nurses. This indicates that, in the context of labour disputes, if there is agreement between the parties that are perceived not to have been fulfilled, this creates an environment favorable to subsequent episodes of strike and protests, which when coincident with PHEICs, exponentiate the risks for all, patients and HCWs.

Both Asian and African countries seem to live in an environment conducive to the appearance of strikes during PHEICs, which only aggravate work conditions that were already difficult, where HCWs already faced a variety of challenges to pursue their professions.

### HCWs’ industrial actions, protests, strikes and lockouts—outputs, outcomes, and impacts.

Results derived from strikes may be analysed from the perspective of the health and care system beneficiaries (the users and patients) (Tables 7 and 8) and from the perspective of the grievances of the HCWs that led to the IAPSLs (Tables 7 and 9).

**Table 7:** Analysis of outputs, outcomes and impact from two different perspectives

Beneficiary	Review questions	Examples		
		Output	Outcome	Impact
Patients and service users	What are the implications of HCWs’ IAPSLs on service delivery, in terms of service disruptions (patients waiting lists), increased mortality and morbidity in in-patient care facilities, temporary and/or permanent drop-out of service, particularly during COVID-19 and other PHEICs? What is the impact of IAPSLs on HCWs, related to the occupations, numbers involved, and days not worked, particularly during COVID-19 and other PHEICs?	Services provided or not provided	Access to care Length of hospital stay Quality of care provided Demand for the care provided	Morbidity, disability and mortality of patients and service users
HCWs	What are the interventions adopted to address the grievances and the demands of IAPSLs, particularly during COVID-19, and other PHEICs; and how the health care workers react to grievances identified and to the interventions implemented to address them?	Agreements achieved in relation to the demands that led to the IAPSLs	Agreements implemented and sustained	Safety, morbidity, disability and mortality of HCWs

**Table 8:** Results observed during the strike from the perspective of service users

Country	PHEIC context	Institutional context/nature of strike	Output	Outcome	Impact	Reference
			Services provided or not provided	Access to care; Length of hospital stay; Quality of care provided; Demand for the care provided	Morbidity, disability and mortality of patients and service users	
Croatia*	SARS threat**	Nationwide physician strike	NA	NA	No impact observed on population-based mortality rate	Erceg et al. [31]
India	COVID-19	Nationwide protest	Disruptions of regular ASHAs (CHWs) duties: attending to pregnant women, providing family planning counselling and supplying contraceptives	Contraceptive demand increased when migrant workers returned home to their villages during lockdown, but contraceptives were only available from the market at high prices—increased demand for ASHAs		Wichterich [32]
Kenya*	Ebola threat**	Data from 4 departments (newborn nursery, paediatric, paediatric surgery, and obstetrics) of not-for profit, faith-based hospital that remined open during physician strike	Public hospitals unable to cope with strike with extra pressure on religious hospital services	Inability of the poorer to pay higher costs in the private sector	Increases in deaths during the strike period across the paediatric, newborn, paediatric surgical and obstetric units with an OR (95% CI) of death of 3.9 (95% CI 2.3–6.4), 4.1 (95% CI 2.4–7.1), 7.9 (95% CI 3.2–20) and 3.2 (95% CI 0.39–27), respectively	Adam et al. [33]
	Ebola threat**	Several strikes by physicians and nurses over several years	During strike periods, the high dependency unit at KCH remained operational but limited paediatric inpatient services were provided. No arrangements were made for hiring replacement staff at KCH during strike periods		In a primary analysis, no change in all-cause population-based mortality was noted during strike periods (adjusted rate ratio [RR] 0.93, 95% CI 0.81–1.08; p = 0.34) when compared with pre-strike period: weak evidence was recorded of variation in mortality rates by age group, with an apparent decrease among infants aged 1–11 months (adjusted RR 0.58, 95% CI 0.33–1.03; p = 0.064) and an increase among children aged 12–59 months (1.75, 1.11–2.76; p = 0.016). No change was noted in mortality rates in post-strike periods and for any category of cause of death	Ong’ayo et al. [35]
	Ebola threat**		Disruptions of service delivery (decline in in-patient admissions, reduction in outpatient service utilisation, households missed care with related health outcomes, delays in accessing care due to uncertainty about which public facilities were open) Alternative sources of care and increased costs of care (private facilities were an alternative source of care, other reported alternatives include use of herbs or self-medication, some households had to borrow or sell household assets to meet costs of care in private facilities) Demotivation among health system staff (health managers reported working long hours and feeling unsupported by their supervisors, service provision was slower and more tasking for the non-striking staff leading to demotivation) Loss of trust in the public health system (service disruption caused by high recurrence of strikes contributed to loss of trust in the public sector among community members)	Reported decline in hospital admissions and theatre services given the interdependence across cadres at hospital level		Waithaka et al. [39]
	COVID-19		Negative impacts on the distribution of LLINs: mass community-based campaigns were delayed; supply chains were affected, leading to temporary declines in routine distributions; disrupted healthcare delivery Supply and COVID-related health system shocks during the first and second waves of infection did not completely interrupt access to LLINs, were temporary and showed signs of resilience by ‘catching up’ on missed distribution opportunities			Suiyanka et al. [38]
	Ebola threat**	Nationwide strikes	Strikes strained relationships and trust between communities and the health system	Access to and utilization of basic maternal and child health services negatively impacted for pregnant women Quality of services decreased The poorest were most affected by the strikes since they were most likely to rely on public services; indirect economic effects due to households having to pay for services in the private sector		Scanlon et al. [36] Scanlon, Maldonado, Ikemeri et al. [37]
	Ebola threat**	Nationwide strikes	Doctors' strike: services were restricted to emergencies; elective procedures were delayed or asked to seek care at private hospitals		There were no significant increases in mortality in the immediate period after the strike compared to baseline period beta (ß) coefficient—7.42 (95% CI − 16.7, 1.85) p = 0.12	Kaguthi et al. [34]
Nigeria	Ebola	Nationwide strikes + local strikes in individual institution	Healthcare delivery disruptions reported	Increase in medical tourism as the richest Nigerians seek health services abroad	Evidence of the impact on poor maternal health indices and variations in infant mortality and in life expectancy during the strikes (26% of the variation in infant mortality and 31% of the difference in life expectancy)	Mayaki and Stewart [41]
	Ebola	National, regional and state-based strikes	Patients’ loss to follow-up	Disruption of patient care unequal access to quality medical care High referral to private hospitals	Increased morbidity and mortality, especially amongst the poor	Oleribe et al. [42]
	Ebola threat**	Local, state and regional; mostly in southern regions	Institutional and service delivery outputs, Professional and training effects	Patients are unable to access health services, including specialised care	Morbidity and mortality impacts (increase in maternal and child mortality, overall mortality and morbidity)	Oleribe et al. [43]
South Africa	COVID-19	General ethical discussion	NA	NA	NA	Mavis Mulaudzi, et al. [44]
South Korea	COVID-19	Data from ED of 1 tertiary-care Academic hospital during physician strike	Shortage of HRH—number of physicians working in the ED reduced to one-third of the usual number Fewer consultations	25% reduction in the total volume of ED visits during the strike Median length of stay in ED decreased from 359 min (interquartile range, IQR: 147–391) in the pre-strike period to 326 min (IQR: 123–318) during the strike period (p < 0.001) median time to admission in ED decreased from 145.5 min (IQR: 83–234.75) to 115 min (IQR: 67.75–191) (p < 0.001)	No significant effect on mortality after adjustments with other variables	Sim Jeongyong, Yuri Choi, Jinwoo Jeong [46]
		6 teaching hospitals		Length of ED stay and the number of patients visiting the ED decreased	No significant in-hospital mortality differences with pre-strike period	Cho et al. [45]
USA	COVID-19	Multiple states				Murphy [47]

*CHWs* Community health workers, *ED* emergency department, *IQR* interquartile range, *NA* Not mentioned

*No cases of Ebola were ever observed in Kenya. No cases of SARS were ever observed in Croatia

**Although the country has no reported cases, the strike took place in the context of a threat from a PHEIC

**Table 9:** Results from the perspective of HCWs

Country	Output	Outcome	Impact	References
	Agreements achieved in relation to the demands that led to the IAPSLs	Agreements implemented and sustained	Safety, morbidity, disability and mortality of HCWs related to the IAPSLs	
India	ASHAs (“voluntary” community healthcare workers) seem to have gained bargaining power from the dependence of the state and the community on their services, but have failed to alter the care extractivism structures (e.g., feminization and informalization of work)		ASHAs were accused of transmitting COVID-19 infections and not wearing protective gear and were attacked, even from family members “high numbers were infected by COVID-19 and died during the pandemic’s first wave”	Wichterich [32]
Kenya	Nurses—Kenya National Union of nurses and government agreed that nurses would receive their nursing service allowance in phases and increase in uniform allowance	Physicians—collective bargaining agreement implemented		Waithaka, Kagwanja, JNzinga et al. [39]

Table 8 reflects a clear impact on outputs and outcomes of services (particularly on the poorest). It also reflects how difficult it is to measure impact on mortality and morbidity unless researchers take either a population-based approach (as done by Erceg et al. [31] in Croatia or a systems approach as done by Adam et al. [33] in Kenya). Cunningham et al. [14] drew attention to the limitations of the use of mortality as outcome, because death can be considered in some way a rare event, and therefore is not a good indicator for changes that do not culminate in death, but that can increase suffering and delays in the use of health services. Smith et al. [54] argue that despite the argument of risks of strikes to patients, there is no clear evidence on an increase in patient morbidity or mortality during periods of strike action. Meanwhile, Friedman et al. [55] showed, based on a population study, that children born during HCWs strikes that took place in Kenya between 1999 and 2014 are more likely to suffer a neonatal death.

Essex, Brophy and Sriram [56] find that most of the strike action occurs in response to structural failings of health systems such as austerity, underinvestment and de-prioritization of health, occupational hierarchies and intersectional power dynamics in health services, and larger societal issues, that are components of health system resilience and sustainability. In light of the recent industrial action in the UK, authors argue that there are risks in failing to strike, insofar as strike action has the potential to change the trajectory of failing healthcare systems. They challenge the framing of strikes as unquestionably harmful to patients, asking the inverse and often overlooked question—that is, how might failure to strike adversely impact patients? Authors argue that when health workers lack other avenues to voice concerns, the failure to strike may actually be more harmful to health in the long run. Also, there is knock-on effects of strike action, particularly in low-resource settings. For instance, increases mortality in nearby facilities dealing with surges in patient pressures which is a reflection of systemic concerns around health service access.

The results must also be interpreted against a background that the PHEIC context itself may have a significant impact on outputs, outcomes, and impacts, and that the strike is a reaction to these rather than just adding to them.

Table 9 reflects the scarcity of evidence found from the perspective of the results achieved (or not) by HCW due to the strikes undertaken. Only in two publications was possible to gather information on this topic.

## Discussion

HCWs’ strikes “create an ethical tension between an obligation to care for current patients (e.g., to provide care and avoid abandonment) and an obligation to better care for future patients by seeking system improvements (e.g., improvements in safety, to access, and in the composition and strength of the health care workforce). This tension is further intensified when the potential benefit of a strike involves occupational self-interest, and the potential risk involves patient harm or death” [57]. But when strikes happen because HCWs protest because salaries are not compatible with the cost of living or the degradation of public health services and underfunding in the health sector, authors such as Smith et al. [54] consider that that there is an ethical concern in the protests. Adobor [58] adds, from a utility-maximizing perspective, that some strikes may be justified if their results mean long-term improvements in the health facilities that serve the majority, being particularly applied in the case of developing countries, considering the fragility of health systems and medical care in these regions, where doctors and other HCWs face the moral dilemma of protecting vulnerable patients or using strikes as a tool to guarantee better health service conditions.

The ethical dimensions of HCWs strikes during COVID-19 was also emphasized in the literature reviewed. An example was the HCWs strike in Hong Kong, where about 8000 HCWs participated in a 5-day strike in early February 2020. Despite the considerable support from public opinion, the strikers were accused of violating professional ethics and of neglecting their accountabilities, which led to moral distress. Evidence indicates that strikers showed care and concern for the safety of the community, sustainability of the health care system, and well-being of all people in Hong Kong [50].

When discussing the ethical dimensions of strikes, the right of populations to receive health care is identified, but it cannot be forgotten that HCWs should be able to work safely and without fear of threats. Thus, without adequate institutional protection, self-protection is justifiable and moral [50].

In this context, Mavis Mulaudzi, et al. [44] critically discussed the ethics of nurses’ choice to strike during the COVID-19 pandemic, and one of the arguments presented is that these HCWs involved in clinical care delivery are most exposed to risks and must be able to express themselves when the conditions for safely performing their work are not guaranteed.

An identical evaluation was made by Murphy [47] about the example of strikes performed by nurses in the USA during COVID-19. The American Nurses Association has published guidelines for working in emergent disasters, including pandemics, based on earlier guidelines by the Institute of Medicine. But a pandemic represents an exceptionality for everyone, including HCWs. The pandemic puts not only patients at risk of infection and possible death, but also HCWs and their families.

According to Murphy [47], what determines the ethics of a strike or work stoppage is the confluence of three factors—the significant risk for HCWs, the duration of this risk, and the ability to mitigate the risk. As a rule, morally, strikes, work stoppages and job reductions by HCWs during disruptive disasters are a wrong choice, as it can put patients at risk. But when rare circumstances are experienced, however, work stoppages can be ethically justified.

The circumstances that may cause a strike in times of pandemic are: HCWs are put at significant risk of infection, damage and even death; the duration of the risk is persistent; inadequate PPE and staff are available to mitigate the risk. Because this means that current patients, future patients, and healthcare and HCWs are placed in an unsustainable and unsafe health care environment [47].

Irimu et al. [59] produced a commentary on HCWs’ strikes that occurred in public health services in Kenya since devolution of healthcare services in 2013. The disproportional effect of the strikes in the poor who are unable to afford private sector alternatives was stressed, raising the ethical dimension of HCWs’ strikes. People's right to health care appears to have been violated to the extent that no public hospital had nearby health services available. This also seems to indicate the need for the specific roles and responsibilities of national and municipal governments to be clearly defined in decentralized health systems.

Kenya is an example of a country where the right to industrial action and strikes was granted but where the institutional mechanisms implemented to manage the labour conflictual environment could be further strengthened. In addition, the specificities of HCWs’ protests in low-income countries with a disproportional impact of the grievances in the poorest population dependent of public health services (ethical dimension of the strike in these cases) should be tackled.

The literature review shows a clear impact on outputs and outcomes of services particularly on the poorest during PHEICs, for instance, when the strikes represent a limitation in terms of access to public health facilities forcing the patients to pay for private services. Similar results were already mentioned as HCWs’ strikes appear to have a greater impact on the poor and vulnerable with no alternative means of obtaining healthcare particularly in countries where provision of healthcare is mostly dependent on public healthcare services [19].

Our review reports that the main reasons for HCWs to undertake a strike are related to the deficiencies of the health systems, which were aggravated in a pandemic context: the significant leadership and governance fragilities of many health and care systems, inappropriate remuneratory systems, health personnel scarcity, inadequate access to medical products and technologies (ventilators, oxygen, and PPE), providing health care in suboptimal working conditions. In India, additionally to the factors mentioned above, the violence against doctors and healthcare professionals that COVID-19 has fueled is mentioned as a motivation for the protests [60].

A study about nurses’ strike in Argentina during COVID-19 [61] also stated that strikes occurred due to work overload and lack of supplies for protection against COVID-19, but the demands exposed historical problems of the profession and more structural problems of the health system (for example, poor working conditions, labor-intensive processes and times, low wages and the consequent permanent lack of personnel to cover the sector's growing demand).

Systematically addressing these challenges, by making adequate investment in education and employment, including decent working conditions, occupational health and safety, fair remuneration, may therefore have the potential to reduce the occurrence of strikes.

These findings have similarities with others previously published pointing as main upstream drivers the rise of consumerism in healthcare, loss of professional autonomy by physicians, many of whom, like other HCWs, work as employees of public or private healthcare organizations with different perspectives of “professionalism” and pressure for unionization [13, 19, 57]. In these organizations pressure for improved performance is escalating “as healthcare institutions attempt to improve the quality of service through restructuring and change, which leads to greater job dissatisfaction, higher turnover, lower morale and increased industrial actions” [62]. Downstream drivers of HCWs’ strikes include failed employer–employee negotiations regarding fair remuneration and benefits, infrastructural deficiencies and inadequate working conditions, concerns about exposure to violence in the workplace and the most diverse policy issues (usually related to workforce development or that hamper the ability of HCWs to carry out their duties effectively) [12, 19, 48]. Interprofessional rivalry for dominance within the hierarchy of healthcare occupations leading the health care system has been cited as a cause of HCWs strikes in Nigeria [19].

Problems in leadership were the cause of protests by HCWs in all the strikes analyzed, those that occurred pre-COVID-19 and during the pandemic. Essentially, there are problems with fulfilling established agreements with health workers, but also a claim to be involved in political decisions and healthcare organization and strategic options. Thus, there seems to be a need to strengthen leadership and management capacities at different levels of health systems (political decision-makers, managers, clinical directors, directors of community health), including through the skills of individual health system leaders and managers, but also in terms of creating or strengthening mechanisms for multi-constituency and intersectoral policy dialogue; processes and mechanisms for addressing labour relations and dispute resolutions may require dedicated attention.

According to several studies [12, 14, 19, 63, 64], the main results of HCWs’ strikes are disruption of healthcare service delivery, leading to cancelation of outpatients' appointments, hospital admissions, and elective procedures and surgeries. Existing evidence suggests that strikes have little impact on in-patient morbidity. There is also no clear evidence of increased in-patients' mortality during strikes, except in isolated cases, where emergency services were also withdrawn during strikes. In some studies readmissions were higher for patients admitted during a strike [33].

The scarcity of population-based analysis to measure impact of HCWs strikes on mortality and morbidity limit the values of these findings as serious morbidity may happen to be transferred to the community rather than taking place in health facilities during strikes.

The literature on the impact of strikes on management reveals “feelings of angst and long hours worked by management”, persisting conflicts and divisions can obstruct teamwork and affect the working environment negatively post-strike. Younger managers, in particular, may need coping support to deal with negative effects on them personally, on their family life and their professional life [10]. In this same direction, Mayaki et al. [41] mentioned the barriers to teamwork including professional hierarchy, role ambiguity, and poor communication as reason to HCWs strikes during PHEICs.

The limited literature suggests the need to invest in measures to prevent strikes, namely respect for direct care providers, a shared governance process, professional autonomy and contribution, and continuous quality improvement [12]. The literature review identified the lack of governance and leadership as one of the reasons to HCWs strikes in Africa and Asia.

In low-income countries, successful resolution of IAPSLs seems easier when “other ministries (finance or public administration ministry) or higher levels of decision-making (such as Prime Minister or President) were involved, rather than the health ministry alone”. Involvement of external international actors in the negotiations is rare, “with the notable exception of human rights nongovernmental organizations (NGOs) in the United Republic of Tanzania in 2012 and Chad in 2018, and the World Bank’s intervention in Guinea Bissau’s health and education workers’ strike” [48].

Despite the recognized feminization of the workforce globally [8], in the review we only found one study [32] that explicitly addressed gender issues as a reason for the strike, the way the pandemic aggravated the vulnerabilities in which women work (low wages, work exposed to violence, poor working conditions and lack of social protection, namely maternity leave). A gender lens is necessary to understand the context, the motivations of health workers' strikes and, above all, the possible need for targeted interventions to meet specific needs.

There are a few limitations in this study. We only identified a limited number of studies from each region, consequently the results might not be generalizable to similar populations in corresponding regions. The reduced amount of literature included in the review did not allow us to fully answer all the initial questions, especially evidence related to the impact of strikes and interventions implemented to respond to the demands of HCWs in strikes.

## Final considerations

IAPSLs by HCWs are not new, but the context of the COVID-19 pandemic has reinforced the need for more in-depth understanding of the phenomenon, with more studies in different countries with distinct health and care systems and interprofessional environments.

The rapid upsurge of the COVID-19 pandemic, like previous PHEICs, aggravated unfavorable working conditions of HCWs both in the Global South and the Global North. These seem to be associated with an above average resort to IAPSLs by HCWs in several countries of the world, with a larger concentration in Asia and Africa.

There is a dispersion in the drivers for the HCWs’ IAPSLs during PHEICs. However, grievances related to medical products and technologies (especially lack of PPE), and financing (disputes due to low wages) seem to stand out. Leadership and governance issues also seem to take on particular relevance as motivations for the protests.

The most common form of IAPSLs reported in the literature during PHEICs are public healthcare sector strikes. The review did not detect any publication on IAPSLs affecting the social care sector. Young HCWs are more prone to resort to strikes when compared to more senior HCWs. This is exemplified in Mexico, where medical students organized a protest during COVID-19 instigated by the death of two young physicians in the course of their work, denouncing "the exploitation of medical health care workers in Mexico" [65]. In India where young physicians went on a strike protesting against an attack on resident doctors in the beginning of COVID-19 pandemic [66]. In addition, in Malaysia, newly qualified doctors organized a nationwide strike in 2021 due to the unequal treatment faced by contractual workers, frustration with the government's lack of long-term solutions, and the enormous burden of the COVID-19 pandemic on this group of precarious workers who were on the front line, and more than half suffered from burnout [67].

It is critical to look at how the decision to go for a strike is conceived in response to HCWs grievances. Is it most common in places where formal associations/unions of HCWs are already well established? Or does the intensity and pattern of strikes among HCWs depend on the governance of health and care institutions and on how they deal with grievances and try to address them before they reach a “striking” point? How important is inter-professional dialogue? And many other issues that need attention as they are not clearly addressed in the literature reviewed. These issues must leave no one behind, ensuring that all categories of HCWs, including less visible categories like CHWs, are considered.

But above all, it is necessary to focus on the preparedness of health and care systems to respond adequately when PHEICs appear. Some of the literature reviewed suggests that if the institutions and health and care systems are not ready, future PHEICs will find HCWs already working dissatisfied and/or at their limit, ready to resort to IAPSLs due to the additional pressures to which they will be necessarily exposed to.

Once a strike begins, it is important to look at the success stories of favorable resolution of strikes among HCWs. What actors were involved and what role did they play? What negotiating processes were considered? What grievances were resolved, and which ones persisted? What contextual factors interfered with the negotiating process? None of the literature selected in this LSR have addressed this.

The main impact of strikes is on the disruption of health care services’ provision. The full impact of the strike on health and care services will not be properly understood unless a system-wide approach is taken, rather than an approach limited to the striking institutions. This is important to allow policymakers to address the health and care needs of the population during the strike in a way that minimizes an additional economic burden to the poorest families that can´t afford to pay private health care when IAPSLs hamper the possibility of access to publicly provided health care.

The full impact of strikes on mortality, disability and morbidity of patients and populations is not possible unless the rates at estimated for whole populations rather than just the population served by the striking institutions, as done by most of the literature reviewed.

When HCWs’ strikes occur in the context of fragile health systems they may jeopardize the fundamental right to health and prevents access to health and care services of the poorest and most vulnerable. Some of the publications discuss the ethical dimensions of HCWs’ strikes in several parts of the world. Ethical tensions escalate when the potential benefit of IAPSLs involve professional self-interest of HCWs (e.g., salary and allowances’ hikes, better working conditions) and the potential risk involves patient neglect, harm or death. It is reported that attitudes of HCWs towards IAPSLs and their ethical concerns differ based on their level of training and their career stage. In the context of a PHEIC HCWs may feel trapped between their ethical obligations towards the persons under their care and their right to claim protection for themselves, from the poor working conditions and lack of PPE, and all the life-threatening risks they face.

Finally, the literature reviewed in the initial phase of this systematic review meets the objective of “understanding the impact of HCWs’ IAPSLs related to the COVID-19 pandemic and to other PHEICs since 2000” but adds little to understand “the relevant interventions to address these IAPSLs” reinforcing the important of regular updates, refining the search terms to allow the identification of literature, if available, that helps to close the knowledge gaps identified. No studies were found in the literature on the analysis of political responses to resolve strikes, the position of the different actors involved in strikes, or negotiation processes between the parties to reach eventual agreements.

### Supplementary Information

Supplementary Material 1.

## Data Availability

The database of the literature review is available upon request from the authors.
