# Supplementary material for: Impacts of industrial actions, protests, strikes and lockouts by health and care workers during COVID-19 and other pandemic contexts: a systematic review
Source: Hum Resour Health. 2024 Jul 2;22:47. doi: 10.1186/s12960-024-00923-y (PMC11221126; doi:10.1186/s12960-024-00923-y)
Supplement: Supplementary file 1 — Supplementary Material 1. [file 12960_2024_923_MOESM1_ESM.docx]

**Supplementary Material**

***Additional file 1 - Eligibility Criteria***

***Additional file 2 – Search strategies***

| **DATABASES** | **SEARCH STRATEGIES** | **N** |
| --- | --- | --- |
| **PUBMED** | ((COVID-19[mj] OR SARS-CoV-2[mj] OR Severe Acute Respiratory Syndrome Coronavirus 2[tiab] OR Coronavirus Disease 2019[tiab] OR 2019 Novel Coronavirus[tiab] OR 2019 New Coronavirus[tiab] OR Wuhan Coronavirus[tiab] OR COVID-19[tiab] OR SARS-CoV-2[tiab] OR 2019-nCoV[tiab] OR HCoV-19[tiab] OR nCoV-2019[tiab] OR Novel Coronavirus 2019-nCoV[tiab] OR Alpha Variant[tiab] OR Beta Variant[tiab] OR Gamma Variant[tiab] OR Delta Variant[tiab] OR Delta Plus Variant[tiab] OR Omicron Variant[tiab] OR Lambda Variant[tiab] OR Influenza A Virus[mh] OR Influenza A Virus*[tiab] OR Influenza Viruses Type A[tiab] OR Middle East Respiratory Syndrome Coronavirus[mh] OR Middle East Respiratory Syndrome Coronavirus[tiab] OR Middle East Respiratory Syndrome Related Coronavirus[tiab] OR MERS[tiab] OR MERS-CoV[tiab] OR Hemorrhagic Fevers, Viral[mh] OR Viral Hemorrhagic Fever*[tiab] OR SARS Virus[mh] OR Severe Acute Respiratory Syndrome Virus[tiab] OR SARS[tiab] OR SARS-COV[tiab] OR Ebola OR "Zika Virus" OR Public Health Emergenc*[tiab] OR Public Health Emergency Preparedness[tiab] OR COVID[tiab] OR Pandemia[tiab] OR Pandemic*[tiab] OR Health Care Provider*[tiab] OR Healthcare Provider*[tiab] OR Healthcare Worker*[tiab] OR Health Care Worker*[tiab] OR Health Care Professional*[tiab] OR Healthcare Professional*[tiab] OR Health Worker*[tiab] OR Health Personnel*[tiab] OR Health Professional*[tiab] OR Physician*[tiab] OR Allied Health Personnel*[tiab] OR Allied Health Professional*[tiab] OR Healthcare Assistant*[tiab] OR Health Care Assistant*[tiab] OR Healthcare Support Worker*[tiab] OR Health Care Support Worker*[tiab] OR Paramedic*[tiab] OR Population Program Specialist*[tiab] OR Community Health Worker*[tiab] OR Community-Based Provider*[tiab]) AND (Industrial Action*[tiab] OR Protest*[ti] OR Lockout*[tiab] OR Strikes, Employee[mh] OR Employee Strike*[tiab] OR Strike*[ti] OR Standstill[ti])) NOT (Letter*[tw] OR Editorial*[tw] OR Release*[tw]) AND (English[lang] OR Portuguese[lang] OR Spanish[lang] OR French[lang] OR Italian[lang] OR Hindi[lang]) AND ("2000/01/01"[PDAT] : "2022/03/01"[PDAT]) | **234** |
| **EMBASE** | ('coronavirus disease 2019'/exp OR '2019 novel coronavirus':ti,ab OR '2019-ncov':ti,ab OR 'covid 19':ti,ab OR 'covid 2019':ti,ab OR 'covid-19':ti,ab OR 'sars coronavirus 2':ti,ab OR 'sars-cov-2':ti,ab OR 'wuhan coronavirus':ti,ab OR 'coronavirus disease 2019':ti,ab OR 'coronavirus infection 2019':ti,ab OR 'ncov 2019':ti,ab OR 'new coronavirus':ti,ab OR 'novel coronavirus 2019':ti,ab OR 'novel coronavirus':ti,ab OR 'severe acute respiratory syndrome 2':ti,ab OR 'covid':ti,ab OR 'Alpha Variant':ti,ab OR 'Beta Variant':ti,ab OR 'Gama Variant':ti,ab OR 'Delta Variant':ti,ab OR 'Delta Plus Variant':ti,ab OR 'Omicron Variant':ti,ab OR 'Lambda Variant':ti,ab OR 'pandemia':ti,ab OR 'pandemic*':ti,ab OR 'severe acute respiratory syndrome'/mj OR 'sars':ti,ab OR 'sars coronavirus infection':ti,ab OR 'sars-cov infection':ti,ab OR 'sars-associated coronavirus infection':ti,ab OR 'sars-related coronavirus infection':ti,ab OR 'severe acute respiratory syndrome':ti,ab OR 'middle east respiratory syndrome'/mj OR 'mers coronavirus infection':ti,ab OR 'mers infection':ti,ab OR 'mers virus infection':ti,ab OR 'mers-cov infection':ti,ab OR 'middle east respiratory syndrome':ti,ab OR 'middle east respiratory syndrome coronavirus infection':ti,ab OR 'middle east respiratory syndrome infection':ti,ab OR 'virus hemorrhagic fever'/mj OR 'epidemic haemorrhagic fever':ti,ab OR 'epidemic hemorrhagic fever':ti,ab OR 'viral haemorrhagic fevers':ti,ab OR 'viral hemorrhagic fevers':ti,ab OR 'virus haemorrhagic fever':ti,ab OR 'virus hemorrhagic fever':ti,ab OR 'influenza a virus (h1n1)'/mj OR 'h1n1':ti,ab OR 'h1n1 influenza a virus':ti,ab OR 'h1n1 influenza virus':ti,ab OR 'h1n1 subtype':ti,ab OR 'h1n1 virus':ti,ab OR 'virus h1n1':ti,ab OR 'ebola hemorrhagic fever'/mj OR 'ebola':ti,ab OR 'ebola fever':ti,ab OR 'ebola hemorrhagic fever':ti,ab OR 'zika fever'/mj OR 'zikv infection':ti,ab OR 'zika fever':ti,ab OR 'zika virus infection':ti,ab OR 'pandemia':ti,ab OR 'pandemic*':ti,ab OR 'health worker*' OR 'healthcare workforce':ti,ab OR 'health workforce*':ti,ab OR 'healthcare worker*':ti,ab OR 'healthcare personnel*':ti,ab OR 'health care professional*':ti,ab OR 'healthcare provider*':ti,ab OR 'health personnel*':ti,ab OR 'healthcare provider':ti,ab OR 'physician*':ti,ab OR 'allied health personnel*':ti,ab OR 'healthcare assistance':ti,ab OR 'paramedic*':ti,ab OR 'population program specialist*':ti,ab OR 'community health worker*':ti,ab OR 'community-based provider*':ti,ab) AND ('industrial action*':ti,ab OR 'protest'/exp OR 'protest':ti OR lockout*:ti,ab OR 'labor strike'/exp OR 'employee strike':ti,ab OR 'employee strikes':ti,ab OR 'general strike':ti,ab OR 'labor strike':ti,ab OR 'labour strike':ti,ab OR 'service strike':ti,ab OR 'worker strike':ti,ab OR strike*:ti OR standstill:ti) NOT (letter*:ti,ab,kw OR editorial*:ti,ab,kw OR release*:ti,ab,kw) AND ([english]/lim OR [french]/lim OR [hindi]/lim OR [italian]/lim OR [portuguese]/lim OR [spanish]/lim) AND [01-01-2000]/sd NOT [01-03-2022]/sd AND [embase]/lim NOT ([embase]/lim AND [medline]/lim) | **99** |
| **SCOPUS** | ALL(COVID-19 OR SARS-CoV-2 OR "Severe Acute Respiratory Syndrome Coronavirus 2" OR "Coronavirus Disease 2019" OR "2019 Novel Coronavirus" OR "2019 New Coronavirus" OR "Wuhan Coronavirus" OR COVID-19 OR SARS-CoV-2 OR 2019-nCoV OR HCoV-19 OR nCoV-2019 OR "Novel Coronavirus 2019-nCoV" OR "Alpha Variant" OR "Beta Variant" OR "Gamma Variant" OR "Delta Variant" OR "Delta Plus Variant" OR "Omicron Variant" OR "Lambda Variant" OR "Influenza A Virus" OR "Influenza Viruses Type A" OR "Middle East Respiratory Syndrome Coronavirus" OR "Middle East Respiratory Syndrome Related Coronavirus" OR MERS OR MERS-CoV OR "Viral Hemorrhagic Fever" OR "SARS Virus" OR "Severe Acute Respiratory Syndrome Virus" OR SARS OR SARS-COV OR "H1N1 Virus" OR Ebola OR "Zika Virus Infection" OR Zika OR "Public Health Emergencies" OR "Public Health Emergency Preparedness" OR COVID OR Pandemia OR Pandemic* OR "Health Care Providers" OR "Healthcare Provider" OR "Healthcare Workers" OR "Health Care Worker" OR "Health Care Professional" OR "Healthcare Professionals" OR "Health Workers" OR "Health Personnels" OR "Health Professionals" OR Physician* OR "Allied Health Personnel" OR "Allied Health Professional" OR "Healthcare Assistant" OR "Health Care Assistant" OR "Healthcare Support Workers" OR "Health Care Support Workers" OR Paramedic* OR "Population Program Specialists" OR "Community Health Workers" OR "Community-Based Providers") AND TITLE("Industrial Actions" OR Protest* OR Lockout* OR "Strikes, Employee" OR "Employee Strikes" OR Strike* OR Standstill) AND (LIMIT-TO(LANGUAGE, "English") OR LIMIT-TO(LANGUAGE, "Spanish") OR LIMIT-TO(LANGUAGE, "French") OR LIMIT-TO(LANGUAGE, "Portuguese")) OR LIMIT-TO(LANGUAGE, "Italian")) OR LIMIT-TO(LANGUAGE, "Hindi")) AND (LIMIT-TO(PUBYEAR, 2000) OR LIMIT-TO(PUBYEAR, 2001) OR LIMIT-TO(PUBYEAR, 2002) OR (LIMIT-TO(PUBYEAR, 2003) OR LIMIT-TO(PUBYEAR, 2004) OR LIMIT-TO(PUBYEAR, 2005) OR (LIMIT-TO(PUBYEAR, 2006) OR LIMIT-TO(PUBYEAR, 2007) OR LIMIT-TO(PUBYEAR, 2008) OR (LIMIT-TO(PUBYEAR, 2009) OR LIMIT-TO(PUBYEAR, 2010) OR LIMIT-TO(PUBYEAR, 2011) OR (LIMIT-TO(PUBYEAR, 2012) OR LIMIT-TO(PUBYEAR, 2013) OR LIMIT-TO(PUBYEAR, 2014) OR (LIMIT-TO(PUBYEAR, 2015) OR LIMIT-TO(PUBYEAR, 2016) OR LIMIT-TO(PUBYEAR, 2017) OR (LIMIT-TO(PUBYEAR, 2018) OR LIMIT-TO(PUBYEAR, 2019) OR LIMIT-TO(PUBYEAR, 2020) OR (LIMIT-TO(PUBYEAR, 2021) OR LIMIT-TO(PUBYEAR, 2022)) | **62** |
| **BVS/LILACS** | (covid-19 OR sars-cov-2 OR "Severe Acute Respiratory Syndrome Coronavirus 2" OR "Coronavirus Disease 2019" OR "2019 Novel Coronavirus" OR "2019 New Coronavirus" OR "Wuhan Coronavirus" OR 2019-ncov OR hcov-19 OR ncov-2019 OR "Novel Coronavirus 2019-nCoV" OR "Influenza A Virus" OR "Middle East Respiratory Syndrome Coronavirus" OR MERS OR "Viral Hemorrhagic Fever" OR "SARS Virus" OR "Severe Acute Respiratory Syndrome Virus" OR SARS OR "H1N1 Virus" OR Ebola OR zika OR "Public Health Emergencies" OR covid OR pandemia* OR pandemic* OR "Sindrome Respiratória Aguda Grave 2" OR "Novo Coronavirus" OR "Alpha Variant" OR "Beta Variant" OR "Gamma Variant" OR "Delta Variant" OR "Delta Plus Variant" OR "Omicron Variant" OR "Lambda Variant" OR "Health Care Providers" OR "Healthcare Provider" OR "Healthcare Workers" OR "Health Care Worker" OR "Health Care Professional" OR "Healthcare Professionals" OR "Health Workers" OR "Health Personnels" OR "Health Professionals" OR physician* OR "Allied Health Personnel" OR "Allied Health Professional" OR "Healthcare Assistant" OR "Health Care Assistant" OR "Healthcare Support Workers" OR "Health Care Support Workers" OR paramedic* OR "Population Program Specialists" OR "Community Health Workers" OR "Community-Based Providers" OR "Prestadores de cuidados de saúde" OR "Trabalhadores de cuidados de saúde" OR "Profissional de cuidados de saúde" OR "Profissionais de saúde" OR "Profissional de saúde" OR "Pessoal de saúde" OR médico* OR "Especialistas em programas de população" OR "Agente de Saúde Comunitário" OR "Provedores baseados na comunidade" OR "Proveedores de atención médica" OR "Trabajadores de la salud" OR "Proveedores comunitarios" OR "Especialistas en programas de población" OR "Oficial de Salud Comunitaria") AND (ti:("industrial action" OR "industrial actions" OR protest* OR lockout* OR "employee strikes" OR strike* OR standstill OR "ações industriais" OR protesto* OR greve* OR paralisação OR "acciones industriales" OR protesta* OR huelga*)) AND (db:("LILACS")) AND (year_cluster:[2000 TO 2022]) | **10** |
| **WHO COVID-19** | (tw:("COVID 19" OR "SARS CoV 2" OR "Alpha Variant" OR "Beta Variant" OR "Gamma Variant" OR "Delta Variant" OR "Delta Plus Variant" OR "Omicron Variant" OR "Lambda Variant" OR "Influenza A virus" OR "Middle East Respiratory Syndrome" OR Coronavirus OR "Hemorrhagic Fevers Viral" OR "SARS Virus" OR Ebola OR "Zika Virus" OR Pandemic* OR "Health Care Providers" OR "Healthcare Provider" OR "Healthcare Workers" OR "Health Care Worker" OR "Health Care Professional" OR "Healthcare Professionals" OR "Health Workers" OR "Health Personnels" OR "Health Professionals" OR physician* OR "Allied Health Personnel" OR "Allied Health Professional" OR "Healthcare Assistant" OR "Health Care Assistant" OR "Healthcare Support Workers" OR "Health Care Support Workers" OR paramedic* OR "Population Program Specialists" OR "Community Health Workers" OR "Community-Based Providers")) AND (tw:("Industrial Actions" OR protest* OR lockout* OR "Strikes, Employee" OR "Employee Strikes" OR strike* OR standstill)) AND db:("GREY-COVIDWHO" OR "PREPRINT-MEDRXIV" OR "COVIDWHO") | **88** |
| **ILO** | (COVID OR Coronavirus OR Pandemic) AND ("Health Personnel" OR "Health Workers") | **99** |
| **OECD** | (COVID-19 OR "Public Health Emergency" OR Pandemic OR Coronavirus OR MERS OR SARS OR Ebola OR "Zika Virus" OR "Influenza A") AND ("Industrial Action" OR Protest OR Lockout OR Strike*) AND ("Health Worker" OR "Health Workers" OR "Health Personnel") | **125** |
| **HSRM** | COVID | **189** |
| **GOOGLE**  **SCHOLAR *** | ("COVID 19" OR "public health emergency") AND ("Health Personnel") AND ("strikes" OR "industrial action" OR "protest" OR "lockout") | **750** |

***Additional file 3 – CAT GRADE CERQual***


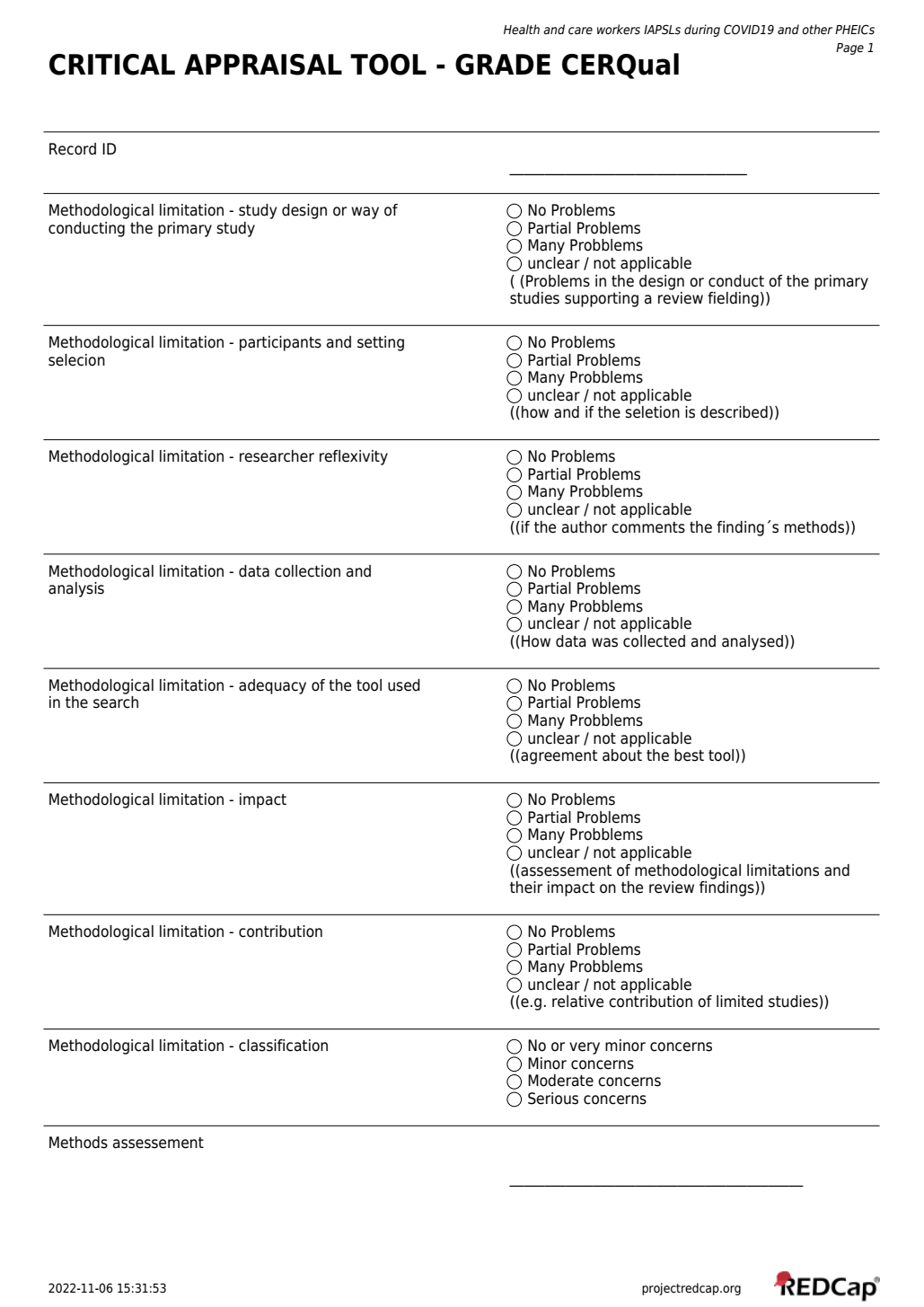


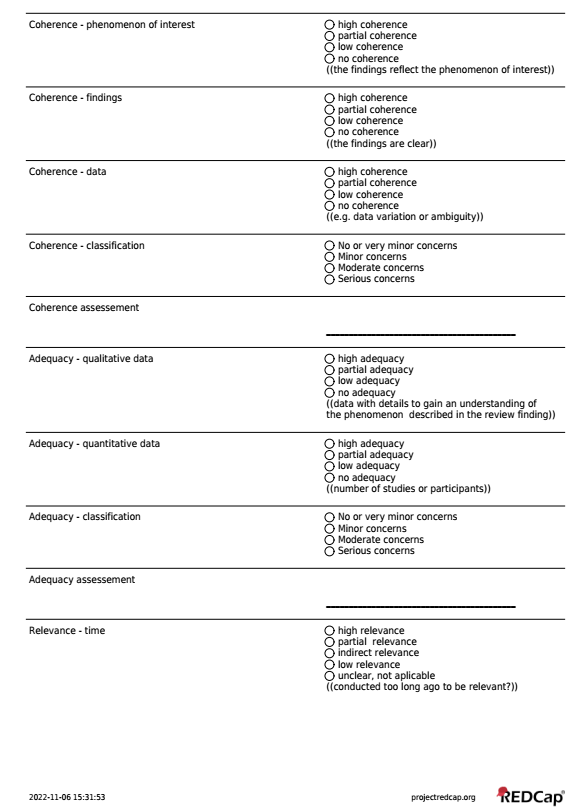


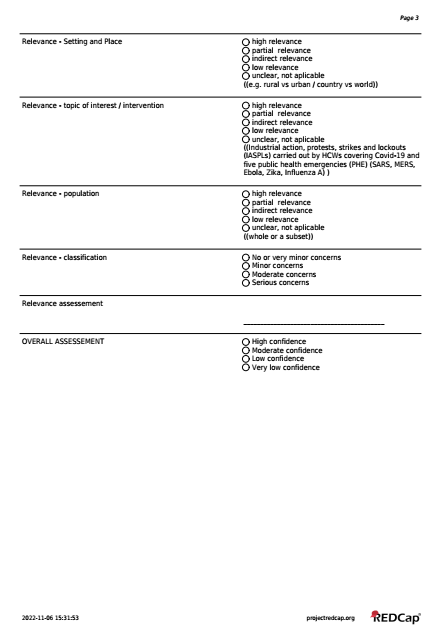


***Additional file 4 – Excluded articles (second phase) and reason for exclusion***

| **Authors** | **Title** | **Reason for exclusion** |
| --- | --- | --- |
| Murphy H. | What will you earn as a junior doctor? | Type of study - Opinion |
| Gabriela Torres-Hernández, Patricio García-Espinosa, Edgar Botello-Hernández, Diego Ortega-Moreno | Mexican Medical Students Protest During COVID-19 Pandemic | Type of study - Opinion |
| Virtual Mentor | Physician activism-doctors go on strike | Type of study (Case) |
| No Author | If pandemic strikes, how much staff will you have? | Type of study - Management case |
| Adelman S. | Physicians for Responsible Negotiation | Not related with phenomenon of interest, e.g., one of the PHEICs |
| Ahmad K. | 200 junior doctors sacked in Zambia | Type of study – News |
| No Author | South Korean doctors end crippling strike | Type of study – News |
| Alubo O, Hunduh V. | Medical Dominance and Resistance in Nigeria's Health Care System | Although the article analyses the phenomenon of interest, generically focused on a broad Ebola context, it is not relevant to answering any of the review questions. |
| Arumugam CT. | COVID 19 - To Strive Against the Strike | Type of study – Commentary |
| J Balen, M. Saddiq, S. Lassa | Nigerian public sector strikes and inter-professional tensions in the time of Ebola - the impact of domestic politics in global health | Type of study – Conference abstract |
| Bowman B. | On the biopolitics of breathing: race, protests, and state violence under the global threat of COVID-19 | Type of study – Article commentary |
| Braithwaite SS. | Collective actions by physicians that do not endanger patients | Not related with phenomenon of interest, e.g., one of the PHEICs |
| Cheng FK. | Ethical Dilemma: An Unprecedented Strike by Health care Workers in Early February 2020 in Hong Kong | Type of study – Personal Reflection |
| Chima SC. | Global medicine: is it ethical or morally justifiable for doctors and other healthcare workers to go on strike? | Not related with phenomenon of interest, e.g., one of the PHEICs |
| Chima SC. | Doctor and healthcare workers strike: are they ethical or morally justifiable: another view | Not related with phenomenon of interest, e.g., one of the PHEICs |
| Crocker K, Cramer B, Hutchinson JM. | Antibiotic availability and the prevalence of pediatric pneumonia during a physicians' strike | Not related with phenomenon of interest, e.g., one of the PHEICs |
| Cruess RL, Cruess SR. | Commentary: professionalism, unionization, and physicians' strikes | Type of study – Commentary |
| Cunningham SA, Mitchell K, Narayan KM, Yusuf S. | Doctors' strikes and mortality: a review | Not related with phenomenon of interest, e.g., one of the PHEICs |
| Davies J. | I'm heartened by the public's support for our summer of protest | Type of study – Commentary |
| Dorozynsk A. | French health staff strike over budget cuts | Type of study – News |
| Dorozynski A. | French healthcare system beset by strikes | Type of study – News |
| Ekoh, P.C., George, E.O. | The Role of Digital Technology in the EndSars Protest in Nigeria During COVID-19 Pandemic | Type of study – Letter |
| Essex R, Weldon SM. | Health Care Worker Strikes and the Covid Pandemic | Type of study – Perspective |
| Falavigna A, da Silva PG. | Brazilian physicians hold national strike against medical insurance companies | Type of study – Fórum |
| Fiester A. | Physicians and strikes: can a walkout over the malpractice crisis be ethically justified? | Not related with phenomenon of interest, e.g., one of the PHEICs |
| Gafni-Lachter L, Admi H, Eilon Y, Lachter J. | Improving work conditions through strike: Examination of nurses' attitudes through perceptions of two physician strikes in Israel | Not related with phenomenon of interest, e.g., one of the PHEICs |
| Gagnon L. | Montreal physicians protest poverty | Type of study – news |
| Goold SD. | Collective action by physicians: beyond strikes | Not related with phenomenon of interest, e.g., one of the PHEICs |
| Darlene R House, Irene Marete, Eric M Meslin | To research (or not) that is the question: ethical issues in research when medical care is disrupted by political action: a case study from Eldoret, Kenya | Type of study – Viewpoint |
| Irimu G, Ogero M, Mbevi G, Kariuki C, Gathara D, Akech S, Barasa E, Tsofa B, English M. | Tackling health professionals' strikes: an essential part of health system strengthening in Kenya | Type of study – Commentary |
| Ismangil, M., & Lee, M. | Protests in Hong Kong during the Covid-19 pandemic | Not related to HCWs |
| Jackson RL. | Physician strikes and trust | Not related with phenomenon of interest, e.g., one of the PHEICs |
| Jansåker F, Holm MKA, Gradel KO, Knudsen JD, Boel JB; Danish Collaborative Bacteraemia Network (DACOBAN). | All-cause Mortality Due to Bacteremia during a 60-Day Non-Physician Healthcare Worker Strike | Not related with phenomenon of interest, e.g., one of the PHEICs |
| Khan Z. | Pakistan's doctors protest at killing of 13 colleagues this year | Type of study – news |
| Kleebauer A. | Staff to vote on 1 per cent rise after last minute talks halt strike | Type of study – news |
| Kloiber O. | The state of the profession - Physicians' strike in Germany | Type of study – Keynote Speech |
| Kmietowicz Z. | Gps shut surgeries in protest at government targets | Type of study – news |
| Kmietowicz Z. | Health workers return medals for Ebola care in protest at UK "hostile environment" policy | Type of study – news |
| Kwon S. | Pharmaceutical reform and physician strikes in Korea: separation of drug prescribing and dispensing | Not related with phenomenon of interest, e.g., one of the PHEICs |
| Li ST, Srinivasan M, Der-Martirosian C, Kravitz RL, Wilkes MS. | Developing personal values: trainees' attitudes toward strikes by health care providers | Not related with phenomenon of interest, e.g., one of the PHEICs |
| Li ST, Srinivasan M, Kravitz RL, Wilkes MS. | Ethics of Physician Strikes in Health Care | Type of study – case |
| Loewy, E. | Of healthcare professionals, ethics, and strikes | Not related with phenomenon of interest, e.g., one of the PHEICs |
| MacDougall DR. | Physicians' strikes and the competing bases of physicians' moral obligations | Not related with phenomenon of interest, e.g., one of the PHEICs |
| Mahase E. | Covid-19: Cases rise in South Korea, China, and Philippines as protests erupt in Thailand | Type of study – news |
| Hospitals & health networks / AHA | Valid protest or ego trip? | Type of study - forum |
| Marchildon, G.P. | Policy lessons from physicians' strikes | Type of study - Commentary |
| Marchildon GP, Schrijvers K. | Physician resistance and the forging of public healthcare: a comparative analysis of the doctors' strikes in Canada and Belgium in the 1960s | Historical perspective |
| Mawere M. | Are physicians' strikes ever morally justifiable? A call for a return to tradition | Not related with phenomenon of interest, e.g., one of the PHEICs |
| Mfutso-Bengu, J.; Muula, A. S. | Is it ethical for health workers to strike? Issues from the 2001 QECH general hospital strike | Type of study - View point |
| Miranda-Chavarría JE. | Huelga de médicos residentes: evidencia de la crisis de valores | Type of study - Letter |
| Mitka M. | Some physicians protest "high-stakes" tests | Type of study - news |
| Muma Nyagetuba JK, Adam MB. | Health worker strikes: are we asking the right questions? | Type of study - Commentary |
| Muula AS, Phiri A. | Reflections on the health workers' strike at Malawi's major tertiary hospital, QECH, Blantyre, 2001: a case study | Not related with phenomenon of interest, e.g., one of the PHEICs |
| Nieto-Gutiérrez W, Bendezú-Quispe G, & Taype-Rondan, A. | ¿Por qué hacen huelga los internos de Medicina en Perú? | Type of study - Letter |
| Njuguna J. | Impact of Health Workers' Strike in August 2014 on Health Services in Mombasa County Referral Hospital, Kenya | Type of study – Brief Communication |
| Pandya SK. | Strikes by physicians in public hospitals in India | Not related with phenomenon of interest, e.g., one of the PHEICs |
| Park JJ, Murray SA. | Should doctors strike? | Type of publication – brief report |
| Parry J. | Hong Kong healthcare workers join protests | Type of study - news |
| Reitemeier PJ. | Collective protest actions by licensed health professionals | Not related with phenomenon of interest, e.g., one of the PHEICs |
| Rivera DA & Adelman S. | Should doctors strike? [2] (multiple letters) | Type of publication – Letter |
| Roberts AJ. | A framework for assessing the ethics of doctors' strikes | Not related with phenomenon of interest, e.g., one of the PHEICs |
| Salazar A, Corbella X, Onaga H, Ramon R, Pallares R, Escarrabill J. | Impact of a resident strike on emergency department quality indicators at an urban teaching hospital | Not related with phenomenon of interest, e.g., one of the PHEICs |
| Sharma DC. | India: health workers strike after attack on junior doctor | Type of study – world report |
| Siegel-Itzkovich J. | Israel's doctors take industrial action | Type of study - news |
| Thompson SL, Salmon JW. | Strikes by physicians: a historical perspective toward an ethical evaluation | Not related with phenomenon of interest, e.g., one of the PHEICs |
| Thompson SL, Salmon JW. | Physician strikes | Not related with phenomenon of interest, e.g., one of the PHEICs |
| Truscott R. | Covid-19: Health worker strikes, limited testing, and clinic closures hamper Zimbabwe's response | Type of study - news |
| Uthayakumar-Cumarasamy A, Sharman M, Calderwood N. | Protest, pandemics and the political determinants of health - the health risks of the UK police, crime sentencing and courts bill 2021 | Not related to HCWs |
| Walsh B, Eskin B, Allegra J, Rothman J, Junker E. | The effects of a physician slowdown on emergency department volume and treatment | Not related with phenomenon of interest, e.g., one of the PHEICs |
| Watts J. | Doctors' first strike in Republic of Korea likely to end | Type of study - news |
| Watts J. | Strikes continue in South Korea as doctors fight drug reform | Type of study - news |
| Watts J. | Korea's pharmacists protest against drug law reforms | Type of study - news |
| Weil LG, Nun GB, McKee M. | Recent physician strike in Israel: A health system under stress? | Not related with phenomenon of interest, e.g., one of the PHEICs |
